# Predicting the role of inequalities on human mobility patterns

**DOI:** 10.1093/pnasnexus/pgaf407

**Published:** 2026-01-20

**Authors:** Alain Boldini, Pietro De Lellis, Salvatore Imperatore, Rishita Das, Luis Ceferino, Manuel Heitor, Maurizio Porfiri

**Affiliations:** Department of Mechanical Engineering, New York Institute of Technology, College of Engineering and Computer Science, PO Box 8000, Northern Boulevard, Old Westbury, New York 11568, USA; Center for Urban Science and Progress, New York University, Tandon School of Engineering, 370 Jay Street, Brooklyn, New York 11201, USA; Department of Mechanical and Aerospace Engineering, New York University, Tandon School of Engineering, 6 MetroTech Center, Brooklyn, New York 11201, USA; Department of Electrical Engineering and Information Technology, University of Naples Federico II, Via Claudio 21, Naples, NA 80125, Italy; Department of Electrical Engineering and Information Technology, University of Naples Federico II, Via Claudio 21, Naples, NA 80125, Italy; Center for Urban Science and Progress, New York University, Tandon School of Engineering, 370 Jay Street, Brooklyn, New York 11201, USA; Department of Mechanical and Aerospace Engineering, New York University, Tandon School of Engineering, 6 MetroTech Center, Brooklyn, New York 11201, USA; Department of Aerospace Engineering, Indian Institute of Science, AE-146, Bengaluru, Karnataka 560012, India; Center for Urban Science and Progress, New York University, Tandon School of Engineering, 370 Jay Street, Brooklyn, New York 11201, USA; Department of Civil and Urban Engineering, New York University, Tandon School of Engineering, 6 MetroTech Center, Brooklyn, New York 11201, USA; Department of Civil and Environmental Engineering, University of California Berkeley, College of Engineering, 760 Davis Hall, Berkeley, CA 94720, USA; Center for Urban Science and Progress, New York University, Tandon School of Engineering, 370 Jay Street, Brooklyn, New York 11201, USA; Marron Institute of Urban Management, New York University, 370 Jay Street, Brooklyn, New York 11201, USA; Center for Innovation, Technology and Policy Research, Technical University of Lisbon, Instituto Superior Técnico, Avenida Rovisco Pais, Lisbon 1049-001, Portugal; Center for Urban Science and Progress, New York University, Tandon School of Engineering, 370 Jay Street, Brooklyn, New York 11201, USA; Department of Mechanical and Aerospace Engineering, New York University, Tandon School of Engineering, 6 MetroTech Center, Brooklyn, New York 11201, USA; Department of Civil and Urban Engineering, New York University, Tandon School of Engineering, 6 MetroTech Center, Brooklyn, New York 11201, USA; Department of Biomedical Engineering, New York University, Tandon School of Engineering, 6 MetroTech Center, Brooklyn, New York 11201, USA

**Keywords:** Inequalities, Migrations, Mobility

## Abstract

Whether in search of better trade opportunities or escaping wars, humans have always been on the move. For almost a century, mathematical models of human mobility have been instrumental in the quantification of commuting patterns and migratory fluxes. Equity is a common premise of most of these mathematical models, such that living conditions and job opportunities are assumed to be equivalent across cities. Growing inequalities in modern urban economy and pressing effects of climate change significantly strain this premise. Here, we propose a mobility model that is aware of inequalities across cities in terms of living conditions and job opportunities. Comparing results with real datasets, we show that the proposed model outperforms the state-of-the-art in predicting migration patterns in South Sudan and commuting fluxes in the United States. This model paves the way to critical research on resilience and sustainability of urban systems.

Significance Statement It is generally understood that economic inequalities and climate change are drivers of human mobility, let that be migration across countries’ borders or commuting patterns within a region. Yet, standard population-level models that describe human mobility do not account for these critical factors. In this article, we propose a new model to describe commuting and migration patterns, explicitly accounting for inequalities between locations. The model is tested on widely different scenarios, outperforming the predictions of state-of-the-art models: migrations in South Sudan, a country torn by a bloody civil war and extreme floods with millions of displaced civilians, and commuting in the United States, where inequalities between cities and rural areas thoroughly affect mobility patterns.

## Introduction

Mobility has always been part of human history (1). Even before the advent of *Homo sapiens*, archaic humans have migrated across continents to find better survival opportunities (2). Migrations have been fundamental for the establishment and development of countries such as the United States (3), with settlers running away from wars, religious intolerance, famines, and extreme poverty (4, 5). In the last couple of decades, we are observing another wave of migrations, both internal (such as from rural areas to cities (6, 7)) and international (including the migrant crisis at the US–Mexico border (8) and the migration routes in the Mediterranean sea (9)). Migrants may not only look for better job opportunities but also flee conflicts, natural hazards, and political persecution and instability. Human mobility is not limited to migrations: with the emergence of large metropolitan areas and mass transit systems during industrialization, people have started commuting daily from their homes in the suburbs to their workplace in city centers (10).

Due to its importance in human existence and history, mobility has been approached by a variety of disciplines beyond geography (11, 12): behavioral ecology, which seeks to interpret human migrations by drawing parallels with animal mobility (13, 14); economics, which analyzes incentives for mobility and their effects on development (15, 16); and, more recently, physics and data science, which leverage the new, large availability of mobility data to build new descriptions of commuting and migration patterns (17, 18). Mathematical models of human mobility help us quantitatively describe migration and commuting patterns, supporting critical decision-making process to prepare for and manage future mobility (19). Building on substantive research in the social sciences highlighting the drivers of human mobility (20–23), several researchers attempted to mathematically predict mobility patterns at different spatio-temporal scales (18, 24–29). One of the earliest population-level description of human mobility is the gravity model (30, 31), which posits the flux from location *i* to location *j* as Newton’s gravitational law, with populations as masses and an inverse dependence on a function $f(r_{ij})$ of the distance $r_{ij}$ between the two locations. Such a phenomenological model incorporates several intuitions regarding mobility: the number of people leaving one location should depend on its population; the attractivity (and therefore the number of people received) of a location should depend on its population; and distance should act as a modulating factor, whereby fluxes should decrease with distance between locations.

Despite good accuracy and widespread use of gravity models (32–35), they suffer from several limitations. First, they fail to capture many features of empirical distributions, such as long-range mobility patterns (36–38). Second, they often require several parameters to fit real datasets (39), hindering their predictive value in new conditions or geographical areas. Third, they may be analytically inconsistent, for example, they do not automatically limit the maximum flux from a location (38). With respect to the latter limitation, microscopic approaches based on entropy maximization (40), random utility maximization (41–44), and information friction (45) can help solve some analytical inconsistencies, but they do not provide functional forms for $f(r_{ij})$ (38).

**Fig. 1.**
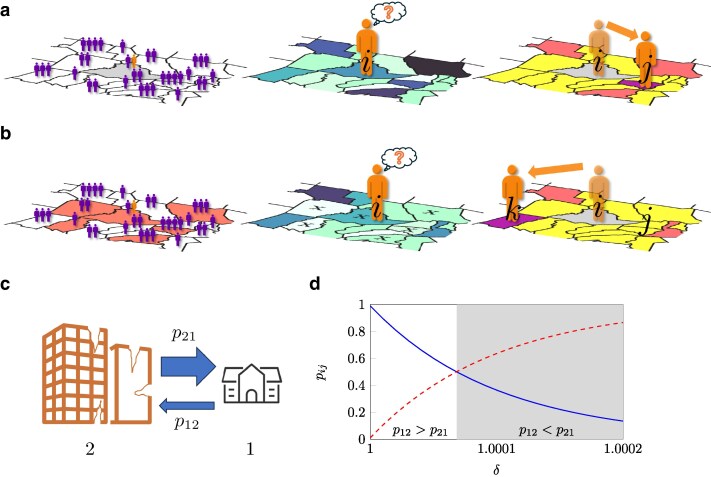
Failure of the standard radiation model to capture the effect of inequalities, and proposed improvement, the modified radiation model, to address this limitation. a) Human decision-making process in the standard radiation model. Opportunities in each location are related to its population (left). When a person (orange) decides to commute or migrate from a region *i* (gray), they set a benefit threshold based on the minimum opportunity they would accept to commute or migrate to another location. This threshold depends on opportunities at the origin *i*. A person evaluates opportunities in all possible destinations (center: a darker color indicates better opportunities for the person at a location; the color of the origin reflects the benefit threshold). Comparing these opportunities with their benefit threshold (right), they determine the regions whose opportunities exceed their threshold. In this way, they differentiate acceptable destinations (dark orange and purple) from unacceptable ones (yellow). The person will then commute or migrate to the closest acceptable destination, in this case *j* (purple). b) Human decision-making process in the modified radiation model. Contrary to the standard radiation model, opportunities in each location vary according to whether the location is suffering from a conflict or not (left; light red regions are part of the conflict). When a person (orange) decides to commute or migrate from a region *i* that is part of the conflict, their benefit threshold is decreased compared to the standard radiation model (center: the color of the origin reflects the benefit threshold). Likewise, opportunities in destinations that suffer from the conflict are reduced compared to the standard radiation model (center: locations that are part of the conflict are marked with a cross; a darker color indicates better opportunities for the person at a location). Comparing opportunities with the benefit threshold (right), the person moves to the closest acceptable destination, in this case *k* (purple), among those where opportunities exceed their threshold (dark orange and purple). Locations that are part of the conflict are more likely to offer opportunities worse than the threshold (yellow), modifying the acceptable destination landscape: for example, *j*, the destination in the standard radiation model, now becomes unacceptable. If the origin is part of the conflict, destinations that would not be acceptable in the standard radiation model can become acceptable: in this example, the destination *k* was unacceptable in the standard radiation model. c) Illustration of the 2-city system: city 1 has a smaller population but is not affected by a conflict, and city 2 has a much larger but affected by a conflict. d) Probabilities $p_{12}$ of migration from city 1 to 2 (blue) and $p_{21}$ of migration from city 2 to 1 (red, dashed) from the modified radiation model (using $n_{1}=10^{4}$ and $n_{2}=10^{6}$), for varying values of the parameter *δ*, which quantifies the inequality between cities 1 and 2. δ=1 corresponds to the standard radiation model, while increasing values greater than 1 indicate a larger penalization of city 2 compared to city 1. The shaded area corresponds to the region of values of *δ* for which we have a higher propensity to move from the larger city 2 to the smaller city 1—a condition unattainable with the standard radiation model.

A strong competitor to gravity models is the *radiation model*, originally proposed by Simini et al. (38). Drawing inspiration from the intervening opportunity model (27, 29) and radiation–absorption models used in physics (46), the radiation model assumes knowledge of the number $T_{i}$ of commuters or migrants from each source location *i*. The model computes the number of them moving to each destination *j*. The individual decision-making process in the standard radiation model is illustrated in Fig. 1a. Each individual who commutes or migrates has a benefit threshold for mobility, which quantifies the minimum “opportunity” a location must offer them for considering to move. The individual moves to the closest destination whose opportunity is higher than their benefit threshold. Benefit threshold and opportunities are computed based on the premise that larger population centers tend to provide more opportunities than smaller ones. Thus, on average, the benefit threshold of a person in a larger city of origin will be higher; likewise, the opportunity of a more populous destination will be larger. The benefit threshold and opportunity are computed from a distribution p(z), by drawing a number of extractions equal to the population of the location and taking the maximum value. In this way, populous cities will be a more desirable choice for human mobility.

This individual-level model can be translated into average fluxes between locations with simple mathematical steps. The flux from location *i* to location *j* (separated by a distance $r_{ij}$) with population $m_{i}$ and $n_{j}$, respectively, is related to the probability $p_{ij}^{R}$ that a commuting or migrating individual from *i* will stop at location *j*,


(1a ) $$T_{ij}=T_{i}p_{ij}^{R},$$

(1b ) $$p_{ij}^{R}=\frac{m_{i}n_{j}}{\left(m_{i}+s_{ij}\right)\left(m_{i}+n_{j}+s_{ij}\right)}.$$

Here, $s_{ij}$ is the population within a radius $r_{ij}$ from the source location *i*, excluding source and destination. The flux in the radiation model is independent of the form of the benefit distribution p(z). The radiation model solves many of the issues of gravity models: it better captures long-range interactions; it is analytically consistent; and it is almost parameter-free (apart from the fraction of mobile population) (38).

The radiation model neglects inequalities between locations associated with living conditions and job quality. We embrace a broad definition of “inequalities” between locations, one that includes the presence of conflicts, natural hazards, and political persecution (47), along with socioeconomic disparities (such as income or wealth inequality) (48–51). All of these variables can affect migration patterns. For example, consider an urban system with only two cities (1 and 2), one much more populous than the other ($n_{2}\gg n_{1}$), see Fig. 1c. Imagine that city 2 is affected by a conflict that poses its residents at risk: would not we expect people from city 2 to flee to city 1? Eq. 1 would suggest the opposite, whereby $p_{12}^{R}=n_{2}/(n_{1}+n_{2})\gg p_{21}^{R}=n_{1}/(n_{1}+n_{2})$. Under the classical radiation model, only the population matters: one needs to account for inequalities across cities to be able to predict a higher tendency to move from 2 to 1, see Fig. 1d. Unfortunately, we have witnessed a similar situation during the ongoing Russian invasion of Ukraine, where over six million Ukrainians flee the country and settled in other nations, such as in Czech Republic (52), whose largest city, Prague, has less than half of Kyiv’s population.

Factors such as conflicts, natural hazards, and socioeconomic inequalities cannot be accounted for in the standard radiation model, where the benefit distribution is the same for all locations. Including inequalities between locations in mobility models is becoming a dire problem, with the increase of people on the move due to wars (53) and climate change (54) and potential increases in disparities in urban economies (55). For example, climate change does not impact all cities equally within the same urban system: some areas may be more affected than others for their specific location (such as coastal regions facing floods (56)) and the economies they have historically pursued (such as agricultural economies facing droughts (57)). Similarly, different cities have reacted differently to technological changes in their economies: Detroit and other cities in the so-called Rust Belt have been severely affected by the decline of US manufacturing (58), while metropolitan areas such as San Francisco and New York have thrived through the information economy (59).

Here, we propose a new mobility model that is intrinsically aware of inequalities among different locations (Fig. 1b). The model assumes that locations can be divided in two or more classes, offering different levels of living conditions and opportunities. The classes have the same shape of the benefit distribution, but the distribution of class *j* is modulated by a scaling factor $\delta_{j}$. Locations are assigned to different classes according to specific features that reflect inequalities, for example, the extent of a conflict. Contrary to other mobility models accounting for variables other than populations (60, 61), we do not alter the final equation of the radiation model in Eq. 1, but modify the human behavior model from which it is derived. With this approach, inequalities are incorporated in a natural way. In contrast to previously proposed schemes, this model does not require to estimate fluxes or add many, noninterpretable parameters—an advantage over microfounded gravity models as well. For the case of a uniform benefit distribution, we find closed-form expressions for the fluxes between locations for an arbitrary number of classes. We present results on migrations in South Sudan, driven by conflicts and natural hazards. We also apply the model to study the effects of socioeconomic inequalities on commuting patterns in the United States, to show how the proposed model outperforms the standard radiation modeling in reconstructing the statistical distribution of mobility patterns in widely different real-world datasets at different time-scales. In addition to the introduction of an inequality-aware model, the main contributions of this study include collecting a dataset about South Sudan, characterized by stark inequalities, and demonstrating the growing importance of factors such as conflicts, natural disasters, and unequal development on mobility patterns.

## Results

### Mathematical model

To simplify the interpretation of formulas, we assume that the *L* locations can be partitioned into two classes, where one class offers worse standard of living conditions and opportunities to their residents than the other (Fig. 1b). The generalization to *C* classes is presented in the Supplementary material. Residents can commute or migrate between any pair of locations, requiring to book-keep four distinct mobility patterns. This is a key difference from the standard radiation model (38) where all locations present equivalent opportunities, so that only one type of mobility pattern shall be resolved.

Within this working assumption, we describe opportunities of locations in each class through different probability density functions $p^{\left(1\right)}(z)$ and $p^{\left(2\right)}(z)$, *z* being the benefit threshold of an individual, equivalent to absorbance in physics. Functions describing classes of locations offering inferior opportunities are more “concentrated” towards the origin to favor the occurrence of worse opportunities. We assume that the second function is a scaled version of the first probability density function, modulated by a parameter *δ*: $p^{\left(2\right)}(z)=\delta p^{\left(1\right)}(z\delta )$.^a^ The case δ=1 corresponds to absence of inequalities, while δ>1 indicates that the second class of locations offers worse opportunities than the first one. The parameter *δ* and the class of each location are assigned based on features that quantify inequalities between locations. While other choices of benefit distributions could be applied to penalize certain locations, the proposed approach allows the formulation of closed-form solutions, a critical aspect for applications in large datasets. We introduce the following notation: $s_{ij}^{\left(k\right)}$ is the population of all locations of class k=1 or k=2 (except of *i* and *j*) within a circle of radius $r_{ij}$ centered at *i*; $s_{ij}$ is the population of all locations of any class (except of *i* and *j*) within a circle of radius $r_{ij}$ centered at *i* (such that $s_{ij}=s_{ij}^{\left(1\right)}+s_{ij}^{\left(2\right)}$); and $c_{i}$ represents the class of the *i*th location.

Our microscale, individual behavioral model is analogous to the absorption–emission model of Simini et al. (38), consisting of two steps. First, we associate with each person *X* who decides to commute or migrate a given location *i* a number $z_{X}$, representing their benefit threshold, computed as the maximum over $m_{i}$ extractions of the corresponding probability density function $p^{\left(c_{i}\right)}(z)$. Second, the surrounding locations have a certain probability to absorb them, according to their size and class. For a generic location *j*, we compute its “opportunity,” analogously to the benefit threshold, by taking $n_{j}$ extractions from the corresponding probability density function $p^{\left(c_{j}\right)}(z)$. The person decides to move to the closest location whose opportunity is greater than its benefit threshold. In this modified model, the benefit distribution is not equal among locations but also depends on the class of the location (i.e. whether the location is disadvantaged with respect to others) and the extent *δ* to which inequality plays a role.

An individual decides to commute or migrate from location *i* to location *j* (i≠j) when *j* is the closest location to *i* that has an opportunity larger than the benefit threshold of that individual. Thus, the probability of one person moving from location *i* to location *j* is


(2) $$\begin{array}{rl} & P\left(1\;|\;m_{i},n_{j},s_{ij}^{\left(1\right)},s_{ij}^{\left(2\right)}\right)\\ & \;=\int_{0}^{\infty }dz\;P_{m_{i}}(z)P_{s_{ij}^{\left(1\right)}}(<z)P_{s_{ij}^{\left(2\right)}}(<z)P_{n_{\;j}}(>z). \end{array}$$

Each term in the integral can be easily interpreted. $P_{m_{i}}(z)$ is the probability that the maximum value extracted from the probability density function corresponding to the origin location ($p^{\left(c_{i}\right)}(z)$) after $m_{i}$ trials is equal to *z*, that is, that a person is assigned a benefit threshold *z*. Following Simini et al., such a quantity is computed as ^b^


(3) $$P_{m_{i}}(z)=m_{i}p^{\left(c_{i}\right)}(<z{)}^{m_{i}}p^{\left(c_{i}\right)}(z),$$

where $p^{\left(c_{i}\right)}(<z)$ represents the cumulative density function of $p^{\left(c_{i}\right)}(z)$ (as all other draws must be below *z* for *z* to be the maximum). Likewise, $P_{s_{ij}^{\left(k\right)}}(<z)$ (k=1 or k=2) is the probability that $s_{ij}^{\left(k\right)}$ numbers extracted from $p^{\left(k\right)}(z)$ are less than *z*: $p^{\left(k\right)}(<z{)}^{s_{ij}^{\left(k\right)}}$. The terms $P_{s_{ij}^{\left(1\right)}}(<z)$ and $P_{s_{ij}^{\left(2\right)}}(<z)$ in the integral require that no location of either class between *i* and *j* offers an opportunity above the benefit threshold *z*, so that the individual would pick that location as their destination. Finally, $P_{n_{\;j}}(>z)$ is the probability that among $n_{j}$ numbers extracted from the probability density function corresponding to the destination location ($p^{\left(c_{j}\right)}(z)$) at least one is greater than *z*,


(4) $$P_{n_{j}}(>z)=1-p^{\left(c_{j}\right)}(<z{)}^{n_{j}}.$$

This final term requires the opportunity of location *j* to be higher than the benefit threshold *z* of the individual, so that the individual would pick *j* as their destination. We acknowledge that this form of lexicographic preference for the destination does not always represent human decision-making, but it offers a simple first-order approximation for the mathematical description of fluxes (18, 38). The integral sums the contributions for any possible benefit threshold extracted.

For each i=1,…,L, we set $p_{ii}=0$ (as a person who commutes or migrate will not stay in the same location) and scale the probabilities in Eq. 2 such that $\sum_{\;j=1}^{L}p_{ij}=1$. The flux $T_{ij}$ from location *i* to *j* is computed by multiplying $p_{ij}$ by the known number of individuals $T_{i}$ from location *i* who commute or migrate.

### Closed-form results for two classes

For the case of two classes “1” and “2,” there are four possible instances of Eq. 2 (1→1, 1→2, 2→1, and 2→2). To perform the computations, we choose uniform distributions, which allow for the formulation of a closed-form solution and represent the simplest ansatz for a benefit distribution, where benefits of commuting and migrating are uniformly distributed among the population. To limit the number of free parameters, we set the first class $p^{\left(1\right)}(z)={\mathrm{Rect}}_{1}(z)$ to be the reference and the second class as $p^{\left(2\right)}(z)=\delta {\mathrm{Rect}}_{\frac{1}{\delta }}(z)$, where ${\mathrm{Rect}}_{a}(z)$ is equal to 1 in [0,a] and zero elsewhere with a>0. From simple algebra, we obtain


(5a ) $$p_{ij}^{\left(2\to 1\right)}=p_{ij}^{R}\delta^{-s_{ij}^{\left(1\right)}}\left[1+\frac{m_{i}+s_{ij}}{n_{j}}(1-\delta^{-n_{j}})\right],$$

(5b ) $$p_{ij}^{\left(2\to 2\right)}=p_{ij}^{R}\delta^{-s_{ij}^{\left(1\right)}},$$

(5c ) $$p_{ij}^{\left(1\to 2\right)}=p_{ij}^{R}\delta^{-m_{i}-s_{ij}^{\left(1\right)}},$$

(5d ) $$\begin{array}{rl} \;p_{ij}^{\left(1\to 1\right)} & =p_{ij}^{R}[\frac{s_{ij}^{\left(2\right)}(m_{i}+s_{ij})\delta^{-m_{i}-n_{j}-s_{ij}^{\left(1\right)}}}{n_{j}\left(m_{i}+n_{j}+s_{ij}^{\left(1\right)}\right)}\\ & \;+\frac{\left(m_{i}+s_{ij})(m_{i}+n_{j}+s_{ij}\right)}{\left(m_{i}+s_{ij}^{\left(1\right)}\right)\left(m_{i}+n_{j}+s_{ij}^{\left(1\right)}\right)}\\ & \;-\frac{s_{ij}^{\left(2\right)}(m_{i}+n_{j}+s_{ij})\delta^{-m_{i}-s_{ij}^{\left(1\right)}}}{n_{j}\left(m_{i}+s_{ij}^{\left(1\right)}\right)}]. \end{array}$$

These expressions are valid for any value of *δ*. If δ=1, the classes are equivalent and all expressions in Eq. 5 reduce to the standard radiation model (38). We have that δ>1 (δ<1) indicates that locations of class “2” offer worse (better) living conditions and opportunities than locations of class “1.” In the Supplementary material, we compare fluxes of standard and modified radiation models for a small network and present results for the limit case δ→∞ (Fig. S1).

The expressions can be simplified for *δ* close to one through linearization, yielding $p_{ij}^{\left(k\to l\right)}=p_{ij}^{R}[1+\rho_{ij}^{\left(kl\right)}(\delta -1)]$, with $\rho_{ij}^{21}=m_{i}+s_{ij}^{\left(2\right)}$, $\rho_{ij}^{22}=-s_{ij}^{\left(1\right)}$, $\rho_{ij}^{12}=-m_{i}-s_{ij}^{\left(1\right)}$, and $\rho_{ij}^{11}=s_{ij}^{\left(2\right)}$. The model and closed-form results can be extended to an arbitrary number of classes, where each class k=1,…,C is assigned a parameter $\delta_{k}$ such that $p^{\left(k\right)}(z)=\delta_{k}p(z\delta_{k})$, being p(z) a reference benefit distribution, see Supplementary material. The linearized model also allows us to provide indications about the choice of the value of $\delta_{k}$, so that the probabilities remain well defined, see Supplementary material.

### Analysis of South Sudan migration data

**Fig. 2.**
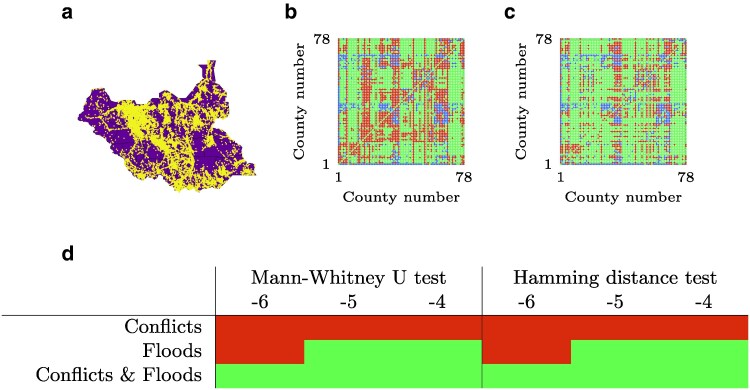
Results for South Sudan. a) Main settlements areas in South Sudan in 2022, highlighted in yellow, based on data from Ref. (65). b, c) Instances where b) the standard radiation model and c) the modified radiation model accounting for both conflicts and floods ($\delta =1+10^{-4}$) overestimate (red), correctly estimate (green), and underestimate (blue) the real migration flows recorded for South Sudan. d) Results of statistical tests for the comparison between the standard and nonlinear modified radiation model with two classes for the South Sudan dataset, for each combination of variable of interest and value of *n* in $\delta =1+10^{n}$. Light green results indicate significance in statistical tests and nonparametric comparisons (α=0.05). See Supplementary material for numerical results (Tables S1–S6).

We study 2020–2021 migrations in South Sudan (Fig. 2a), a country plagued by civil war and environmental disasters such as floods, causing 2 million people to migrate internally over the past few years and 2.3 million refugees in other countries (62). This is an original dataset that we collected and studied for the first time in the present work. Raw data from public sources have been polished, pre-processed, and made available to the scientific community in a dedicated online repository (63).

As we anticipate large inequalities between counties, we utilize the nonlinear version of our model with two classes. The classes are selected depending on casualties in conflicts, scaled over the population, and intensity of floods in the counties (see Materials and methods). Specifically, we explore three different possibilities to assign counties to the penalized class (δ>1). Under the first option, penalized counties are the 15% with higher casualties per capita. Under the second option, the penalized class includes the 15% of counties that are mostly affected by floods. Under the third option, the penalized class is the union of the sets of penalized counties in the first two options. The choice of 15% as the threshold for the most affected counties is a trade-off between two contrasting requirements: (i) enough counties should be affected to achieve statistical differences; and (ii) only a handful of counties are likely to be affected so severely as to obtain considerable changes in migration patterns. In the Supplementary material (Tables S7–S10), we present a sensitivity analysis of the results with respect to this penalization threshold, highlighting the robustness of results to changes in the selection of this parameter. We repeat the analysis for different values of *δ* ($1+10^{n}$, n=−6,−5,−4) for the penalized class. We compare model predictions with those from the standard radiation model. For both the models, we assume that $T_{i}$ is known a priori, such that we only redistribute the overall flux from *i* across all possible destinations, and we round fluxes to the closest integer.

For this and the following dataset, we consider the application of both population-level models (the standard and modified radiation models) for the statistical reconstruction of the mobility fluxes. Thus, we present a comparison between the standard and modified radiation models via statistical tests that allow us to understand which of the two models better reconstructs some statistical features of mobility. For this reason, we propose nonparametric tests that do not introduce any hypotheses on relationships between variables.

For each estimated matrix of fluxes, we compute the error distribution by taking the absolute value of the difference between the estimated and real matrix of fluxes. We utilize two tests to assess whether the modified radiation model outperforms the standard radiation model. We employ the Mann–Whitney *U* test (MW) (64) to test the null hypothesis that the absolute errors’ distribution are statistically the same, with the alternative hypothesis being that the absolute errors of the modified radiation model are statistically lower than those of the standard radiation model. To provide an indication of whether the structure of the data generated by the models is similar to that of the actual fluxes, we use the Hamming distance (that is, the number of nonzero elements). This choice is motivated by the fact that most fluxes are zero, as most commuting and migration patterns are limited to neighboring locations. Specifically, we compute the Hamming distance for the error distributions of both modified and standard radiation models and statistically compare them (see Materials and methods). We regard the MW and Hamming distance tests to be better indicators of the quality of the model than other indicators, such as least squares, root mean square, or the coefficient of determination, as they do not assume any relationship for the data (in particular, they are apt for nonlinear relationships) and better capture whether the results reconstruct the overall structure of the mobility patterns. To exclude the possibility that improved model predictions are related to more parameters in the modified model, we employ a nonparametric test that penalizes the additional parameters in the model (see Materials and methods). Specifically, we check whether a random assignment of the counties into two classes yields different predictions than assignment based on conflicts and floods. To further explore the role of the additional parameters in our modified model, we replicate a statistical test with the Akaike information criterion (see Supplementary material, Table S11). Only when statistics are significant and pass the nonparametric test (so that considered inequality variables actually provide information about mobility patterns), we consider the modified radiation model to outperform the standard one. For all tests, we use a significance level α=0.05.

In Fig. 2d, we show the results of the comparisons of the modified radiation model against the standard one. We observe that conflicts, individually, cannot explain the recorded migration patterns. Only when considering floods or combining conflicts and floods we obtain significant results for both statistical tests and nonparametric comparisons. These results point at a critical role of climate and the environment on mobility. Further, we fail to identify significant results for individual variables for small values of *δ* for the penalized class, indicating that large penalties for locations affected by conflicts and floods are necessary.

Figure 2b,c displays the comparison between the errors made by the standard radiation model and those of the modified radiation model considering both conflicts and floods, with $\delta =1+10^{-4}$, against real flows. Most of the fluxes among counties are small (zero or a few individuals, especially at larger distances), such that both models are able to correctly estimate a good fraction of the fluxes. We find that the proposed model considerably increases the number of correctly estimated fluxes (in this case, 4,050 against 3,366 from the standard model), especially by reducing fluxes that are overestimated. In the Supplementary material, we present a rank comparison between fluxes of standard and modified radiation models against real fluxes (Fig. S2). The Supplementary material also contains additional analyses based on a weighted Hamming distance, $F_{1}$-score on the error, and Spearman correlation coefficient (Tables S12–S16). These additional results show that, although the modified radiation model provides a better statistical reconstruction of the fluxes, it does not fully capture the complexity of migratory fluxes in South Sudan.

Overall, modeling of the South Sudan dataset provides interesting insights into the migration patterns in the country. We found that conflicts alone are not enough to describe the overall migration patterns. There are a few potential motivations for this observation. First, we averaged casualties from the conflict over several years and considered a single point in time for migrations. It is likely that the civil war caused fast migrations, which may not have been permanent. The main phases of the civil war concluded in 2018, with residual communal clashes and violence against civilians to these days. Return migration over subsequent years—not accounted neither in the standard nor in the modified models—could partially explain the lack of significance for conflicts alone. Also, the effects of the more recent floods on migration in the country are likely stronger than conflicts. Major floodings occurred in South Sudan since 2019, closer in time to the recordings of migratory fluxes (2020–2021). Recent flooding may have caused even more damage than the conflict, by completely curtailing the sources of sustenance of the local population. Second, we cannot exclude that the recording of migration data in South Sudan is not completely accurate, as it is based on limited survey data. Yet, the dataset employed in these analyses is the best available estimate, given the challenging conditions in South Sudan.

Another important result for the South Sudan dataset is the limited improvements in other performance metrics, such as the weighted Hamming distance and the Spearman correlation coefficient (see Supplementary material). Both the standard and modified radiation models cannot completely capture the decision-making process in extreme conditions, within a war-torn country affected by natural disasters. For example, people tend to move more to South Sudan’s capital, Juba, than either model predicts. Although migrations to the capital may be overpresented in the dataset, it is likely that radiation models struggle to fully capture mobility patterns in extreme circumstances.

### Analysis of US commuting data

As a second example of application of our model, we study commuting fluxes between US counties from 2011 to 2015. This dataset has been often analyzed in the context of population-level models, and in particular for the original radiation model (38). This example is considered to assess the performance of the proposed modified radiation model at different time scales, beyond long-term migrations. While the original radiation model is able to statistically capture the features of this dataset, we investigate whether inequalities among locations can help us improve on the reconstruction of the statistical distribution of commuting fluxes. We examine a set of variables that are proxies of factors that reflect inequalities (66), namely: (i) Gini index (GI); (ii) poverty ratio (PR); (iii) ratio between median rent and median household income (RAT); and (iv) unemployment rate (UR) (see Materials and methods). For all variables, we consider averages over the 5 years.

As we expect smaller differences from the fluxes of the radiation model (due to the smaller effect of socioeconomic variables on migrations compared to conflicts and natural hazards) and larger variability in the variables of interest (due to the large number of counties) compared to the South Sudan dataset, we utilize the linearized model with an arbitrary number of classes. A higher value of each of the socioeconomic variables is a proxy of higher inequality and, possibly, of worse job opportunities. Thus, we set the number of classes equal to the number of counties whose variable is above the 90% percentile, plus one. We assign to all counties with a value of the variable below the 90% percentile to the first class, with $\delta_{1}=1$. For all the remaining counties, we set a value $\delta_{k}$ that varies linearly with the distance from the 90% percentile, between 1 (assigned to the county with the variable equal to the 90% percentile) and 1+Δ (assigned to the county with the maximum value of the variable). In this case, the choice of the 90% percentile as a threshold is guided by the consideration that socioeconomic inequalities are likely to disrupt commuting patterns only in extreme conditions (unlike conflicts and natural hazards).

For both the standard and modified radiation models, we compute $T_{i}$ from the population $m_{i}$ of each county using a linear regression of real commuting data. We find that 7.895% of the population commutes outside their county of residence ($T_{i}=0.0785\;m_{i}$). We assign to *Δ* all powers of 10 between $10^{-8}$ and $10^{-6}$. We utilize the same tests as those in the South Sudan case to compare the standard and modified radiation models (see Materials and methods).

**Fig. 3.**
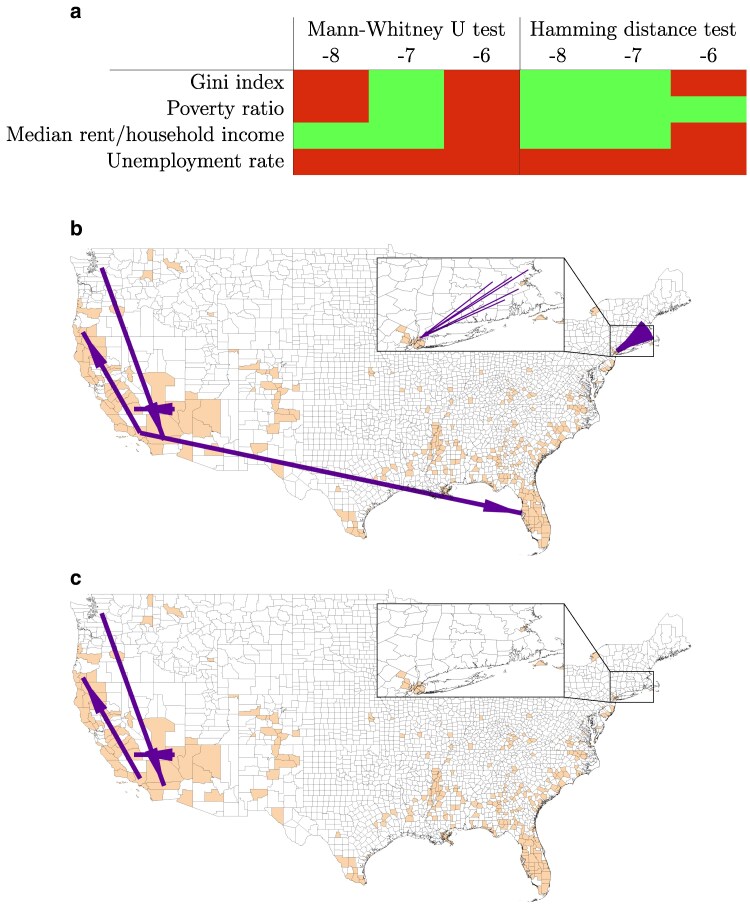
Results for the United States. a) Results of statistical tests for the comparison between the standard radiation model and linearized version of our model for the US dataset, for each combination of variable of interest and value of *n* in $\Delta =10^{n}$. Light green results indicate significance in statistical tests and nonparametric comparisons (α=0.05). See Supplementary material for numerical results (Table S17–S22). b, c) Errors in fluxes for b) the standard radiation model and c) modified radiation model with RAT variable in US commuting patterns ($\Delta =10^{-7}$). Only the 10 fluxes that are changed the most in percentage by the modified model are shown. In c), lack of arrows which were present in b) indicate zero errors compared to real fluxes. All displayed fluxes are overestimated compared to real fluxes. Orange counties correspond to the largest 10% RAT values. The inset shows the area surrounding New York City.

Figure 3a shows the results of statistical tests comparing the linearized modified radiation models against the standard one. For moderate values of *Δ* ($10^{-7}$), modified models using GI, PR, and RAT outperform the standard radiation model, regardless of the statistical test. Interestingly, UR does not improve our predictions of commuting patterns, regardless of *Δ*. It is tenable that differences in unemployment rates do not considerably affect commuting patterns, as in the short-term employment shocks are absorbed locally (67) and long-term variations often result in migrations (68). Small ($10^{-8}$) and large values ($10^{-6}$) of *Δ* may hinder significant differences. For small *Δ*-s, effects may be too small to produce a significant difference, at least for MW tests. For large *Δ*-s, the hypotheses of the linearized model are presumably not satisfied. In the Supplementary material, we introduce additional analyses based on a weighted Hamming distance, $F_{1}$-score, and Spearman coefficient (Tables S23–S27). The $F_{1}$-score confirms the results on the main manuscript. The weighted Hamming distance and Spearman coefficient analyses corroborate the importance of the poverty ratio in shaping commuting patterns in the United States. Further, these two analyses show a benefit in accounting for unemployment rates, possibly pointing at more complex effects of this variable than the one considered in the main manuscript.

In Fig. 3b,c, we show the errors in the 10 fluxes that change the most in percentage values between the standard radiation model and modified radiation model using RAT and $\Delta =10^{-7}$. The size of the arrows is proportional to the error made in predicting commuting fluxes. We opted to show the largest percentage changes rather than the largest absolute changes in the models as the latter occur for large commuting fluxes. Both models do not account for local features that contribute to shape the largest fluxes, thus both retaining large errors. Orange counties show the 10% of counties with the largest RAT values, indicating the counties that are penalized in the modified model. The standard radiation model overestimates several long-range commuting patterns from or to counties with high RAT, for example between Los Angeles and St. Petersburg in Florida and from Rhode Island/Massachusetts to counties forming New York City (Fig. 3b). The modified radiation model correctly estimates most of these fluxes (as they are zero or comprising only a few individuals), removing overestimates in long-range commuting patterns that are absorbed by closer locations that are not affected by large RAT. These results highlight how the modified model helps better reconstruct the statistical distribution of commuting fluxes.

## Discussion

Mobility is a fundamental process in biological and nonbiological systems, from morphogenesis of cells (69) and animals’ seasonal migrations (70), to the motion of bacteria (71) and colloids (72) through chemical gradients. Collective movements may support the emergence of higher-level, functional structures (such as embryos from cells (73)) or enable better exploitation of available resources (such as herds of animals migrating for food and reproduction (74)). Human mobility is among the most complex of mobility processes (75). Humans not only migrate long distances to settle where they can improve their living conditions and escape conflicts and natural disasters but also commute daily for work and leisure (18). The complexity of human mobility stems from the multitude of economic, social, and environmental factors underlying individual decision-making.

Current mathematical models of human mobility do not explicitly account for inequalities in living conditions and job opportunities. However, wars, natural hazards, political persecutions, and socioeconomic disparities have always been fundamental drivers of human mobility (51). With the increase in global conflicts (53), environmental crises from climate change (54), and potentially increasing inequalities in urban economies (55), there is a dire need for inequality-aware mobility models. To address this critical limitation, we propose a population-level model of human mobility, derived from first principles, that can account for inequalities that drastically affect human decision-making on mobility. We show that the proposed model can outperform the state-of-the-art radiation model in predicting migrations in South Sudan and commuting patterns in the United States, modulating fluxes (especially long-range ones) from or to disadvantaged locations. We focus on statistical comparisons about the structure of the commuting patterns, without introducing additional hypotheses about relationships between variables.

The model contains few additional parameters that modulate the mobility patterns ($\delta_{k}$ and the threshold to distinguish disadvantaged locations from nondisadvantaged ones). We do not use optimization or fitting algorithms to select these parameters, as we are guided by order of magnitude arguments (see Supplementary material) as well as simple empirical considerations. For example, we select the threshold based on the need to include a sufficient number of disadvantaged locations, while retaining only locations for which the penalty effect is strong enough to see an effect, and a linear relationship between relevant inequality variables and penalties of different classes for $\delta_{k}$ as a simple ansatz. Yet, we acknowledge that the addition of parameters introduces additional complexity and arbitrariness. We addressed these concerns in several ways. First, we performed a nonparametric test to compare improvements in results when parameters are chosen according to relevant variables against instances where parameters are chosen randomly. If choosing parameters according to relevant variables does not significantly improve results against randomly assigning the parameters, we consider the model to not outperform the standard radiation model. Second, we replicated one of the statistical tests with an explicit penalization of the additional parameters by using the Akaike information criterion, obtaining analogous results (see Supplementary material, Table S11). Finally, we showed that our results are robust to a change in the threshold with a robustness analysis (see Supplementary material, Tables S7–S10). Overall, the proposed model is able to better capture statistical features of the mobility patterns in the two considered datasets.

The proposed model suffers from two main limitations: its results depend on the benefit distribution p(z) considered and its derivation from simplified human behavior assumptions, common to radiation models. While a uniform distribution with finite support allows us to establish analytical results, other distributions may better capture benefit distributions, depending on proxies used to measure benefit. One of such examples includes Pareto distributions (76), which capture the distribution of income. Pareto distributions comprise rare but very extreme events, thus possibly increasing some long-range fluxes that can only be absorbed by the largest cities with the best opportunities (77). Other distributions can be used to model tax brackets, accessibility of amenities, and other features important for decision-making in mobility. Thus, different distributions may capture inequalities at an individual level (78, 79), on top of inequalities between locations. These and other nonrectangular p(z) are adapt to numerical implementations of the modified radiation model. A potential avenue of future work could include a general pipeline to account for inequalities directly from data, for example through the estimation of probability density functions via kernel density estimation (80). The modifications introduced in this manuscript can be also applied to more advanced radiation models (81, 82). Other fundamental hypotheses of the proposed model are the lexicographic preferences for the destination location and the form of the scaling of the benefit distribution for disadvantaged locations. While these choices may not always accurately represent human decision-making in mobility processes, we decided to retain them, together with uniform distributions, to guarantee the formulation of closed-form solutions for the fluxes. We consider closed-form solutions fundamental to reduce the computational complexity of the model, especially in view of the large datasets associated with mobility patterns (which scale quadratically with the number of locations). The proposed benefit distribution scaling approach is not the only pathway to introduce asymmetries between locations. For example, one could introduce a posteriori parameters that modulate the fluxes to and from selected locations (similar to previous approaches that used features other than population (60)), or modify the whole benefit distribution at certain locations. The advantage of our approach stands in the combination of a few features: (i) the ability to compute closed-form solutions; (ii) the interpretability of the model in terms of human behavior at the microscopic scale; and (iii) the limited number of additional parameters in the model.

The second limitation of the model stands in its simplified assumptions of human behavior common to all radiation-like models—namely, the selection of a threshold and lexicographic preference for the closest destination that satisfies the threshold. These assumptions restrict the use of the proposed model to relatively large spatial scales, where simplifications represent statistical averaging of individual behaviors (analogous to mean-field approximations in statistical physics). Similar to the standard radiation model, our approach considers population-level features of mobility, and seeks to reconstruct statistical features of migration and commuting patterns, rather than precise numerical values for all the fluxes. Absolute errors for both models for individual fluxes may be large, as local factors are not accounted for in the models. Yet, we showed that our inequality-aware model provides a better statistical description of mobility patterns than the standard radiation model.

A critical application of the proposed model is the study of inequalities associated with climate change, which will likely cause unprecedented environmental migrations in the next decades (83, 84). This topic is the object of intense political debate (85, 86) and is in dire need of scientifically backed evidence. The study of the South Sudan dataset points at a crucial role of climate inequalities on human migrations associated with exposure to floods. At the moment, the field lacks reliable datasets that track where people moves in the aftermath of extreme events (and if and how they return) (87–89). The South Sudan dataset, which we collated, is a first step to bridge this knowledge gap. We anticipate that new datasets, along with the proposed model, will help predict the effect of climate change on future human migrations. Such predictions are of critical importance for cities, the most sought-after destinations of migrants (90, 91). These analyses can equip cities’ governance with adequate information tools to prepare for stresses on their infrastructural and service systems.

## Materials and methods

### Data of South Sudan migrations

South Sudan gained independence from Sudan in July 2011. In December 2013, a civil war broke out, and the conflict officially ended in September 2018 with the signing of the “Revitalized Agreement on the Resolution of the Conflict in South Sudan” (R-ARCSS) (92). Five years of war forced nearly 4.2 million people to flee their homes in search of safety. Of these 4.2 million people, about a half remained in the country—so called Internally Displaced People (IDPs), according to the United Nations (UN).^c^ In 2020, 1.6 million of these IDPs were still displaced, mainly because of communal clashes and other forms of violence against civilians. In 2021, such a figure further increased, to about 2 million people, partly due to floods that occurred in the country since 2019; for example, 835,000 people were affected by flooding between May and December 2021 (94).

#### Migration data

The International Organization for Migration (IOM) collects data about displaced people in South Sudan through the Displacement Tracking Matrix (DTM) system, encompassing multiple datasets (95). To study internal movements in the country, we used the “Flow Monitoring” dataset, which provides quantitative estimates of the flow of individuals through specific locations. In particular, the Flow Monitoring Registry (FMR) surveys people on the move during selected hours of observations at key transit points (Flow Monitoring Points [FMPs]), within South Sudan and on its borders. FMR datasets are organized by month starting from 2020, and for each observed displacement they include the following information:

- Name and position of the FMP where the data was collected;
- Total number of individuals in the surveyed group, and number of those who declare South Sudanese nationality;
- Type of travel relative to South Sudan (incoming, outgoing, internal, or transit);
- Country of departure and (intended) destination, including levels 1 and 2 administrative sub-areas;
- Camp of departure or destination, if any;
- Time spent by the group at the location of departure and intended time to be spent at the destination, used to distinguish medium/long term displacements from others;
- Whether or not the destination matches the group’s habitual residence;
- Reason for displacement, differentiating between voluntary and forced ones; and
- Means of transport.

We used data from January 2020 to December 2021. Note that data in FMR mostly refer voluntary, short-term migrations, even though some forced migrations are still registered. Moreover, FMR data only capture the volume and characteristics of the flows transiting through the FMPs, and they do not provide a full or statistically representative picture of internal and cross-border movements in South Sudan.

#### Population data

In the absence of a recent census, we used estimates of the South Sudan population annually as part of the Humanitarian Programme Cycle analysis carried out by the UN High Commissioner for Refugees. These estimates are developed and endorsed by the Common Operational Datasets for population statistics (COD-PS), and disseminated through the OCHA’s Humanitarian Data Exchange website (HDX) (65). CODs are the reference datasets to support operations and decision-making in the initial response to a humanitarian emergency. Only estimates of South Sudanese nationals in South Sudan are included in the COD-PS dataset, thereby excluding refugees and asylum seekers in and out of the country. In this work, population estimates in 2020 were linked to the administrative boundaries (AB) in the COD-AB dataset, also available at the HDX website (65).

In our work, we also utilized settlements data at the subnational level to obtain more accurate information about the population distribution in the country. These data are also available at the HDX website, and they comprise geographical coordinates of settlements with their administrative sub-areas from level 1 to 3.

#### Natural disasters and conflicts

As we intended to model the impact of natural disasters and conflicts on internal migrations, we also considered two datasets on flooding and violence in South Sudan. With respect to flooding, for the year 2021, we employed the OCHA dataset on the number of people affected by floods at the county level. For 2020, data on flooding were not available from HDX, so an estimate was obtained from Figure 1 in Ref. (94) that visualizes the number of people affected by floods in each county with a resolution of 25,000 people. We chose the middle value of the county bin as an estimate of the number of affected people.

With respect to conflicts, data were collected from the Armed Conflict Location & Event Data Project (ACLED) (96), which reports information about the type, agents, location, date, and other characteristics of political violence events around the world. In this work, we focused on the number of casualties in South Sudan due to violence against civilians at the level of the county with a resolution of 1 year. We aggregated values over 2020 and 2021 and scaled the number of casualties by the population, to obtain a relative probability of civilians being affected by the conflict.

#### Preprocessing of the data

A county-level origin–destination (OD) matrix is built by using movements described in FMR data. The ones that are internal to South Sudan are extracted from the available datasets and only South Sudanese nationals who moved from one county to another are taken into account, filling the element of the matrix corresponding to their reported origin and intended destination. A single OD matrix is obtained by adding the displacements that occurred in all months of 2020 and 2021. In this case, 965 nonzero flows are present.

Settlements data and COD-AB boundaries are used to compute the geographical coordinates of the centroid of each county. In particular, settlements within the boundaries of each county are extracted and their coordinates are averaged to compute the centroid of that county.

### Statistical test for South Sudan

We utilize the MW statistical test to compare the results of the standard radiation model against the modified radiation model utilizing different variables of interest and values of *Δ*. The null hypothesis of the test is that the absolute errors’ distributions for standard and modified radiation models are statistically indistinguishable. On the other hand, the alternative hypothesis requires the absolute errors of the modified radiation model to be statistically lower than those of the standard radiation model.

We seek to assess whether the improvement in the MW test is only related to a higher number of parameters (*δ* and the percentile of the distribution of variables that separates the two classes of locations). To this end, we compare the results of the modified radiation model against an empirical distribution of the same statistics. To generate this empirical distribution, we generate fluxes via the modified radiation model with the same *δ*, with classes assigned randomly while maintaining the same number of counties in each class. We utilize these fluxes to perform a MW statistical test against the standard radiation model. We repeat this procedure 1,000 times to generate a distribution of statistics from the test. Next, we perform a nonparametric test by comparing the statistics from the test with the modified model with relevant variables against the 5% percentile of the empirical distribution.

We repeat the same procedure for the test on the Hamming distance on the error between each model and real fluxes. To assess whether the improvement in the Hamming distance in the modified radiation model is significant, we utilize a statistical test. We model the Hamming distance as the outcome of random extractions from a binomial distribution over $N^{2}$ trials (being *N* the number of counties), with success probability estimated from the Hamming distance for the standard radiation model. The null hypothesis is that the Hamming distance observed in the modified radiation model is generated from the same binomial distribution of the standard one. The alternative hypothesis is that there are two different binomial distributions generating the data, where the success probability of the binomial distribution for the modified radiation model is lower than that of the standard one. We use as *P*-value for the test the probability that the Hamming distance is equal or smaller than that observed in the modified radiation model (that is, the cumulative binomial distribution), assuming the success probability to be equal to that estimated from the standard radiation model.

To penalize the additional parameters in the model, we compare Hamming distances from modified radiation models against an empirical distribution of Hamming distances. Similar to the previous test, we generate fluxes from the modified radiation model by assigning counties to the two classes randomly, while maintaining the same ratio of locations in each class. Next, we compute the Hamming distance for the error between these fluxes and the real fluxes. Repeating the procedure 1,000 times, we generate the empirical distribution of Hamming distances, from which we extract the 5% percentile, which we use to perform a nonparametric comparison.

### Data of commuting patterns and socioeconomic variables for the United States

Commuting fluxes between US counties from 2011 to 2015 are available from the American Community Survey (97). The variables utilized as proxies of socioeconomic inequalities, available from the Agency for Healthcare Research and Quality (98), are defined as follows:

- Gini index (GI): Gini index of income inequality (99). It is a measure of income inequality based on the Lorenz curve, which plots the cumulative percentage of the overall income earned by the bottom x% of the population in terms of income. For a perfectly equally distributed income, the curve would be a straight, 45° line, called line of equality. In practice, the real curve always lays below the line of equality. The Gini index is the ratio between the area between the line of equality and the Lorenz curve, over the entire area below the line of equality. The index goes from a minimum of 0 (the entire income is concentrated in a single individual) to a maximum of 1 (the income is equally distributed among all individuals).
- Poverty ratio (PR): Percentage of population with income to poverty ratio under 0.50. The poverty line is defined by US Census Bureau ($27,479 for a family of four in 2021 (100)). Higher values indicate larger portions of the population below the poverty line.
- Ratio between the median rent and median household income (RAT): Ratio between the annualized median gross rent (dollars) and the median household income (dollars, inflation-adjusted to data file year). Higher values indicate that residents pay a larger amount of the household income in rent.
- Unemployment rate (UR): Unemployment rate per 100 population (ages 16 and over).

The plots of the counties over the 90% percentile for GI, PR, RAT, and UR are available in the Supplementary material (Fig. S3).

### Statistical tests for the United States

We replicate the same statistical tests proposed for South Sudan in the case of US counties, with a few differences. First, in the MW tests, as the vast majority of commuting fluxes are zero, we remove from the distributions the values for which both the errors of the standard and modified radiation models are zero to emphasize the differences between the methods. Second, we limit random extractions in the nonparametric tests to 200, due to the large number of counties (generation of the random dataset employed about a week on a standard laptop).

## Supplementary Material

pgaf407_Supplementary_Data

## Data Availability

The dataset on South Sudan is available at https://osf.io/3fzrx/?view_only=fb3b84a9aa03487eb6baaa99b6d9a6cb (63).

## Notes

a This implies that the respective cumulative density functions satisfy $P^{\left(2\right)}(z)=P^{\left(1\right)}(z\delta )$.

b This expression is easily derived by using the cumulative density function $P_{m_{i}}(<z)$, which is equal to $(p^{\left(c_{i}\right)}(<z){)}^{m_{i}}$ due to the independence of the draws. By recalling that the probability density function is equal to the derivative of its cumulative density function with respect to its argument, the claim follows.

c UN defines IDPs as “persons or groups of persons who have been forced or obliged to flee or to leave their homes or places of habitual residence, in particular as a result of or in order to avoid the effects of armed conflict, situations of generalized violence, violations of human rights or natural or human-made disasters, and who have not crossed an internationally recognized state border” (93).

## Preprints

A preprint of this article is published at https://doi.org/10.48550/arXiv.2412.13331.
