# Supplementary material for: Predicting the role of inequalities on human mobility patterns
Source: PNAS Nexus. 2026 Jan 20;5(1):pgaf407. doi: 10.1093/pnasnexus/pgaf407 (PMC12817215; doi:10.1093/pnasnexus/pgaf407)
Supplement: pgaf407_Supplementary_Data [file pgaf407_supplementary_data.pdf]

PNAS Nexus, YEAR, pp. 1–16

doi: DOI HERE
Advance Access Publication Date: Day Month Year
Paper

PAPER

Supplementary Material - Predicting the role of inequalities on human mobility patterns

Alain Boldini,[a,b,c] Pietro De Lellis,[d] Salvatore Imperatore,[d] Rishita Das,[b,c,e] Luis Ceferino,[b,f,g] Manuel Heitor[b,h,i] and Maurizio Porfiri[b,c,j,*]

[a]Department of Mechanical Engineering, New York Institute of Technology, College of Engineering and Computer Science, PO Box 8000, Northern Boulevard, Old Westbury, 11568, New York, United States, [b]Center for Urban Science and Progress, New York University, Tandon School of Engineering, 370 Jay Street, Brooklyn, 11201, New York, United States, [c]Department of Mechanical and Aerospace Engineering, New York University, Tandon School of Engineering, 6 MetroTech Center, Brooklyn, 11201, New York, United States, [d]Department of Electrical Engineering and Information Technology, University of Naples Federico II, Via Claudio 21, Naples, 80125, NA, Italy, [e]Department of Aerospace Engineering, Indian Institute of Science, AE-146, Bengaluru, 560012, Karnataka, India, [f]Department of Civil and Urban Engineering, New York University, Tandon School of Engineering, 6 MetroTech Center, Brooklyn, 11201, New York, United States, [g]Department of Civil and Environmental Engineering, University of California Berkeley, College of Engineering, 760 Davis Hall, Berkeley, 94720, California, United States, [h]Marron Institute of Urban Management, New York University, 370 Jay Street, Brooklyn, 11201, New York, United States, [i]Center for Innovation, Technology and Policy Research, Technical University of Lisbon, Instituto Superior Técnico, Avenida Rovisco Pais, Lisbon, 1049-001, Portugal and [j]Department of Biomedical Engineering, New York University, Tandon School of Engineering, 6 MetroTech Center, Brooklyn, 11201, New York, United States

[*]To whom correspondence should be addressed: mporfiri@nyu.edu

Draft

Comparison on a small graph

We illustrate how the proposed model differs from the standard radiation model on a small graph, composed of ten nodes with a random position in $[0, 1] \times [0, 1]$ and random population (Fig. S1). We arbitrary label the nodes of the graph and assign the first three of them to the "2" class, to signify that they are experiencing detrimental effects from a variable (for example, they have higher unemployment rates). The rest of the nodes are assigned to the "1" class.

We run a series of simulations by varying the parameter δ. Specifically, we consider the values $\delta = 1$ (equivalent to the standard radiation model), 1.01, and 1.05, corresponding to increasing inequities between affected and unaffected locations, and we assume that 25% of the population moves from each node. Population fluxes for different values of δ are shown in Fig. S1(b-d).

The standard radiation model (Fig. S1b) shows large fluxes between the closest communities, that is, the pairs $(2, 6)$ and $(4, 7)$, with a proportional bias toward the larger community of the pair, 6 and 7, respectively. All of the nodes display a sizeable flux toward 1, which is the largest and most central community.

Upon disrupting communities 1, 2, and 3, we discover substantial changes in mobility patterns. Already for $\delta = 1.01$ (Fig. S1(c)) we find a decrease in all the fluxes from the rest of the nodes to these communities. We still register a consistent flux from 3 to 1, due to the relative isolation of 3 and the large size of 1. We find a considerable increase in the flux from 1 to the closest non-affected community, 5, and to 6, which is considerably larger than 5, although a bit further. In addition, migration fluxes from 2 to 6 become much larger, but there is still a considerable number of migrants from 6 to 2, despite the inequalities in 2. Fluxes between unaffected nodes are also affected. Most notably, we find larger fluxes from community 6 to 5, likely redirected away from 1 and 2, and between 6 and 7.

For $\delta = 1.05$ (Fig. S1(d)), we find that almost all fluxes from unaffected communities to affected ones are almost negligible. Interestingly, there is still a significant flux from node 3 to 1. Despite the disruption in 1, most of the migrants from 3 still decide to move to 1, as there is no closer unaffected location (see Section 2). Unexpectedly, we find that some fluxes from affected communities to unaffected ones increase, while others decrease. In particular, while for smaller values of δ we record an increase of the fluxes from 1 to 5 and from 1 to 6, at larger values we find that the flux from 1 to 5 still increases, while that from 1 to 6 decreases. In fact, for large δ-s, the population in a disadvantaged location tends to migrate only to the closest, unaffected location, regardless of the population, as they seek to get away in the fastest possible way from the affected location (see Section 2). Fluxes

© The Authors. All rights reserved.

from unaffected locations 6 and 7 shift from the affected nodes 1 and 2 to the closest unaffected ones, 4 and 5, and long-range migration patterns between 6 and 7 are further strengthened.

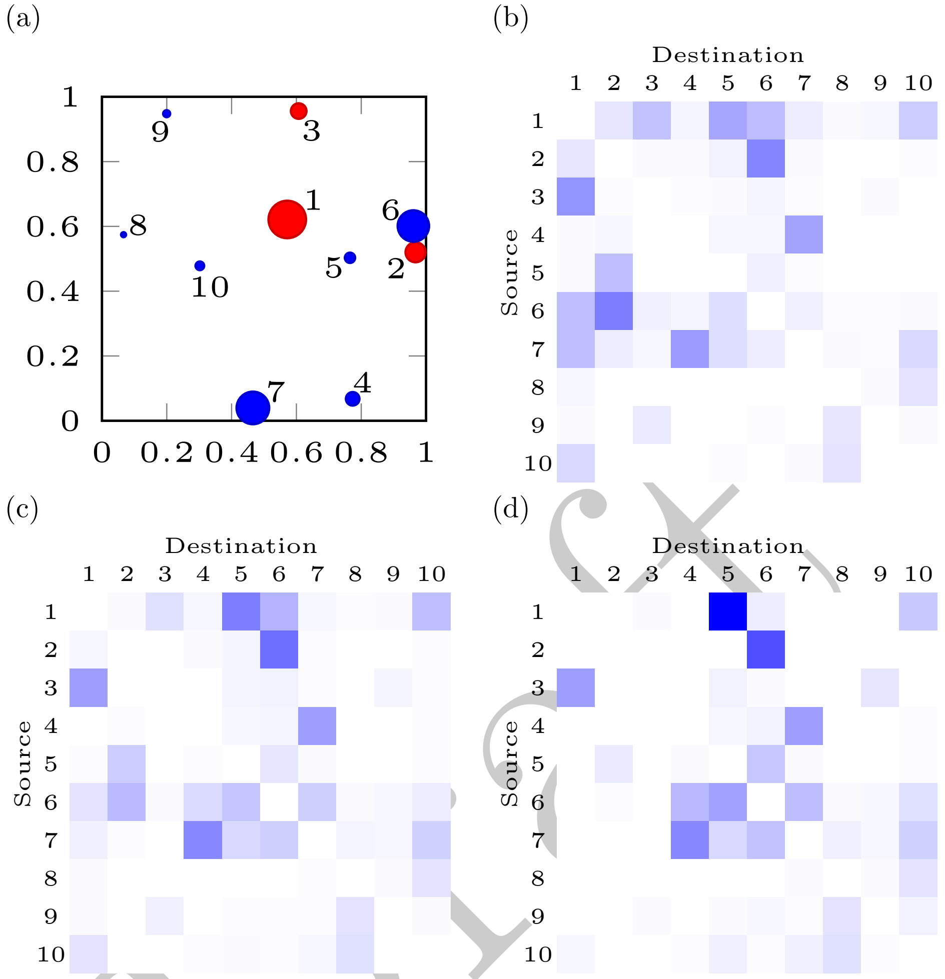

Fig. S1. Comparison of the proposed model and standard radiation model on a small graph. (a) Spatial location and labeling of the nodes, whose size is proportional to the population (red: "2", blue: "1"). (b-d) Population fluxes between nodes for $\delta = 1$ (equivalent to the standard radiation model), 1.01, and 1.05, respectively; larger population fluxes correspond to darker shades, with an equal color scale among the three panels.

Limit of the model for $\delta \to \infty$

An interesting case for our model is that in which $\delta \to \infty$, which corresponds to a case in which inequalities are so stark that affected locations basically offer no opportunity and do not attract any migrant or commuter. In this case, for the nonlinear model with two classes and arbitrarily large δ, we find

$$p_{ij}^{(2\to 1)} = \begin{cases} 0 \ \text{ if } s_{ij}^{(1)} > 0, \\ \frac{m_i}{m_i + s_{ij}^{(2)}} \ \text{ if } s_{ij}^{(1)} = 0, \end{cases} \quad , \tag{1a}$$

$$p_{ij}^{(2\to 2)} = \begin{cases} 0 \ \text{ if } s_{ij}^{(1)} > 0, \\ \frac{m_i n_j}{\left(m_i + s_{ij}^{(2)}\right)\left(m_i + n_j + s_{ij}^{(2)}\right)} \ \text{ if } s_{ij}^{(1)} = 0, \end{cases} \quad , \tag{1b}$$

$$p_{ij}^{(1\to 2)} = 0, \tag{1c}$$

$$p_{ij}^{(1\to 1)} = \frac{m_i n_j}{\left(m_i + s_{ij}^{(1)}\right)\left(m_i + n_j + s_{ij}^{(1)}\right)}. \tag{1d}$$

We find that the unaffected locations follow the standard radiation model as if there was no affected location. There is no flux from unaffected to affected locations. In affected locations, the entire population is emitted. We find that an affected location

only have non-zero fluxes to locations within the radius of the closest unaffected location. Within that radius, affected locations exchange fluxes between themselves following the standard radiation model. Thus, small, isolated, disadvantaged communities surrounded by other affected locations would still migrate to communities with larger populations, even if disadvantaged as well. The rest of the population is absorbed by the closest unaffected location.

Nonlinear model for an arbitrary number of classes

We present the derivation of our model for an arbitrary number of classes. To find closed-form expressions, we use uniform distributions, such that $p^{(c_i)}(z) = \delta_{c_i}\mathrm{Rect}_{\delta_{c_i}^{-1}}(z)$ and the corresponding cumulative mass function is

$$P^{(c_i)}(< z) = \delta_{c_i}\mathrm{Ramp}_{\delta_{c_i}^{-1}}(z), \tag{2}$$

with

$$\mathrm{Ramp}_{\delta_k^{-1}}(z) = \begin{cases} z, & 0 \leq z < \delta_k^{-1} \\ \delta_k^{-1}, & z \geq \delta_k^{-1} \end{cases} . \tag{3}$$

We establish

$$\begin{aligned} P\left(1|m_i, n_j, s_{ij}^{(1)}, \ldots, s_{ij}^{(C)}\right) &= \int_0^\infty \mathrm{d}z P_{m_i}(z) \prod_{k=1}^{C} \left[P_{s_{ij}^{(k)}}(< z)\right] P_{n_j}(> z) \\ = m_i\delta_{c_i}^{m_i} \prod_{k=1}^{C} \delta_k^{s_{ij}^{(k)}} &\int_0^\infty \left[\mathrm{Ramp}_{\delta_{c_i}^{-1}}(z)\right]^{m_i} \mathrm{Rect}_{\delta_{c_i}^{-1}}(z) \left[\mathrm{Ramp}_{\delta_k^{-1}(z)}\right]^{s_{ij}^{(k)}} \mathrm{d}z \\ - m_i\delta_{c_i}^{m_i}\delta_{c_j}^{n_j} \prod_{k=1}^{C} \delta_k^{s_{ij}^{(k)}} &\int_0^\infty \left[\mathrm{Ramp}_{\delta_{c_i}^{-1}}(z)\right]^{m_i} \mathrm{Rect}_{\delta_{c_i}^{-1}}(z) \left[\mathrm{Ramp}_{\delta_k^{-1}(z)}\right]^{s_{ij}^{(k)}} \left[\mathrm{Ramp}_{\delta_{c_j}^{-1}(z)}\right]^{n_j} \mathrm{d}z. \end{aligned} \tag{4}$$

Without lack of generality, let us define $\delta_1 < \delta_2 < \cdots < \delta_C$. We first focus on the first term. By carrying out the integration, we write

$$\begin{aligned} &m_i\delta_{c_i}^{m_i} \prod_{k=1}^{C} \delta_k^{s_{ij}^{(k)}} \int_0^\infty \left[\mathrm{Ramp}_{\delta_{c_i}^{-1}}(z)\right]^{m_i} \mathrm{Rect}_{\delta_{c_i}^{-1}}(z) \left[\mathrm{Ramp}_{\delta_{c_k}^{-1}(z)}\right]^{s_{ij}^{(k)}} \mathrm{d}z \\ &= m_i\delta_{c_i}^{m_i} \prod_{k=1}^{C} \delta_k^{s_{ij}^{(k)}} \left[\frac{\delta_C^{-(m_i+s_{ij})}}{m_i + s_{ij}} + \sum_{r=c_i}^{C-1} \frac{1}{\prod_{s=r+1}^{C} \delta_s^{s_{ij}^{(s)}}} \frac{\delta_r^{-\left(m_i+\sum_{t=1}^{r} s_{ij}^{(t)}\right)} - \delta_{r+1}^{-\left(m_i+\sum_{t=1}^{r} s_{ij}^{(t)}\right)}}{m_i + \sum_{t=1}^{r} s_{ij}^{(t)}}\right]. \end{aligned} \tag{5}$$

Next, we focus on the second term. Assuming $c_i < c_j$, the integration provides

$$\begin{aligned} &m_i\delta_{c_i}^{m_i}\delta_{c_j}^{n_j} \prod_{k=1}^{C} \delta_k^{s_{ij}^{(k)}} \int_0^\infty \left[\mathrm{Ramp}_{\delta_{c_i}^{-1}}(z)\right]^{m_i} \mathrm{Rect}_{\delta_{c_i}^{-1}}(z) \left[\mathrm{Ramp}_{\delta_k^{-1}(z)}\right]^{s_{ij}^{(k)}} \left[\mathrm{Ramp}_{\delta_{c_j}^{-1}(z)}\right]^{n_j} \mathrm{d}z \\ &= m_i\delta_{c_i}^{m_i}\delta_{c_j}^{n_j} \prod_{k=1}^{C} \delta_k^{s_{ij}^{(k)}} \left[\frac{\delta_K^{-(m_i+n_j+s_{ij})}}{m_i + n_j + s_{ij}} + \sum_{r=c_j}^{C-1} \frac{1}{\prod_{s=r+1}^{C} \delta_s^{s_{ij}^{(s)}}} \frac{\delta_r^{-\left(m_i+n_j+\sum_{t=1}^{r} s_{ij}^{(t)}\right)} - \delta_{r+1}^{-\left(m_i+n_j+\sum_{t=1}^{r} s_{ij}^{(t)}\right)}}{m_i + n_j + \sum_{t=1}^{r} s_{ij}^{(t)}}\right. \\ &\left. + \sum_{r=c_i}^{c_j-1} \frac{1}{\prod_{s=r+1}^{C} \delta_s^{s_{ij}^{(s)}}} \frac{\delta_r^{-\left(m_i+\sum_{t=1}^{r} s_{ij}^{(t)}\right)} - \delta_{r+1}^{-\left(m_i+\sum_{t=1}^{r} s_{ij}^{(t)}\right)}}{m_i + \sum_{t=1}^{r} s_{ij}^{(t)}}\right]. \end{aligned} \tag{6}$$

For $c_i \geq c_j$, the third term in parentheses is zero, while the summation in the second term starts from c_i.

Merging the results in (5) and (6), we find

$$\begin{aligned} p_{ij}^{c_i \to c_j} = m_i\delta_{c_i}^{m_i} \prod_{k=1}^{C} \delta_k^{s_{ij}^{(k)}} &\left[\frac{\delta_C^{-(m_i+s_{ij})}}{m_i + s_{ij}} - \delta_{c_j}^{n_j} \frac{\delta_K^{-(m_i+n_j+s_{ij})}}{m_i + n_j + s_{ij}}\right. \\ &+ \sum_{r=c_i}^{C-1} \frac{1}{\prod_{s=r+1}^{C} \delta_s^{s_{ij}^{(s)}}} \frac{\delta_r^{-\left(m_i+\sum_{t=1}^{r} s_{ij}^{(t)}\right)} - \delta_{r+1}^{-\left(m_i+\sum_{t=1}^{r} s_{ij}^{(t)}\right)}}{m_i + \sum_{t=1}^{r} s_{ij}^{(t)}} \\ &- \delta_{c_j}^{n_j} \left(\sum_{r=c_j}^{C-1} \frac{1}{\prod_{s=r+1}^{C} \delta_s^{s_{ij}^{(s)}}} \frac{\delta_r^{-\left(m_i+n_j+\sum_{t=1}^{r} s_{ij}^{(t)}\right)} - \delta_{r+1}^{-\left(m_i+n_j+\sum_{t=1}^{r} s_{ij}^{(t)}\right)}}{m_i + n_j + \sum_{t=1}^{r} s_{ij}^{(t)}}\right. \\ &\left.\left. + \sum_{r=c_i}^{c_j-1} \frac{1}{\prod_{s=r+1}^{C} \delta_s^{s_{ij}^{(s)}}} \frac{\delta_r^{-\left(m_i+\sum_{t=1}^{r} s_{ij}^{(t)}\right)} - \delta_{r+1}^{-\left(m_i+\sum_{t=1}^{r} s_{ij}^{(t)}\right)}}{m_i + \sum_{t=1}^{r} s_{ij}^{(t)}}\right)\right]. \end{aligned} \tag{7}$$

Draft

Linearized model for an arbitrary number of classes

Due to the complexity of the formula in (7), we put forward a linearized version that is fully transparent and interpretable. We linearize the first term in (5) for $\delta_k \to 1$, $k = 1, \ldots, C$. The zero-th order term is $m_i/(m_i + s_{ij})$. The first-order terms in δ_k depend on the relation between δ_k and δ_{c_i}. We have that, for $\delta_k \neq \delta_{c_i}$, the first-order terms are in the form

$$\frac{m_i}{m_i + s_{ij}} s_{ij}^{(k)} (\delta_k - 1). \tag{8}$$

If instead $\delta_k = \delta_{c_i}$, we find the first-order term is

$$\frac{m_i}{m_i + s_{ij}} (s_{ij}^{(c_i)} - s_{ij})(\delta_{c_i} - 1). \tag{9}$$

We can rewrite the linearization as

$$\begin{aligned} & m_i \delta_{c_i}^{m_i} \prod_{k=1}^{C} \delta_k^{s_{ij}^{(k)}} \int_0^\infty \left[\mathrm{Ramp}_{\delta_{c_i}^{-1}}(z)\right]^{m_i} \mathrm{Rect}_{\delta_{c_i}^{-1}}(z) \left[\mathrm{Ramp}_{\delta_{c_k}^{-1}(z)}\right]^{s_{ij}^{(k)}} \mathrm{d}z \\ & \approx \frac{m_i}{m_i + s_{ij}} \left[1 + \sum_{k=1}^{C} s_{ij}^{(k)} (\delta_k - 1) - s_{ij} (\delta_{c_i} - 1)\right]. \end{aligned} \tag{10}$$

We then linearize the second term in (6). The zero-th order term of the sum is $m_i/(m_i + n_j + s_{ij})$. Similar to the previous case, the first order terms depend on the relation between δ_k, δ_{c_i}, and δ_{c_j}. For $\delta_k \neq \delta_{c_i}$ and $\delta_k \neq \delta_{c_j}$, the first-order terms are in the form

$$\frac{m_i}{m_i + n_j + s_{ij}} s_{ij}^{(k)} (\delta_k - 1). \tag{11}$$

For $\delta_k = \delta_{c_i}$,

$$\frac{m_i}{m_i + n_j + s_{ij}} (s_{ij}^{(c_i)} - s_{ij} - n_j)(\delta_{c_i} - 1). \tag{12}$$

For $\delta_k = \delta_{c_j}$,

$$\frac{m_i}{m_i + n_j + s_{ij}} (s_{ij}^{(c_j)} + n_j)(\delta_{c_j} - 1). \tag{13}$$

Then, we write the linearization as

$$\begin{aligned} & m_i \delta_{c_i}^{m_i} \delta_{c_j}^{n_j} \prod_{k=1}^{C} \delta_k^{s_{ij}^{(k)}} \int_0^\infty \left[\mathrm{Ramp}_{\delta_{c_i}^{-1}}(z)\right]^{m_i} \mathrm{Rect}_{\delta_{c_i}^{-1}}(z) \left[\mathrm{Ramp}_{\delta_k^{-1}(z)}\right]^{s_{ij}^{(k)}} \left[\mathrm{Ramp}_{\delta_{c_j}^{-1}(z)}\right]^{n_j} \mathrm{d}z \\ & \approx \frac{m_i}{m_i + n_j + s_{ij}} \left[\sum_{k=1}^{C} s_{ij}^{(k)} (\delta_k - 1) - (s_{ij} + n_j)(\delta_{c_i} - 1) + n_j (\delta_{c_j} - 1)\right]. \end{aligned} \tag{14}$$

From the sum of (10) and (14), we retrieve from simple algebra

$$p_{ij}^{(c_i \to c_j)} = p_{ij}^{\mathrm{R}} \left[1 + \sum_{k=1}^{C} s_{ij}^{(k)} (\delta_k - 1) + m_i (\delta_{c_i} - 1) - (m_i + s_{ij})(\delta_{c_j} - 1)\right], \tag{15}$$

which is equivalent to

$$p_{ij}^{(c_i \to c_j)} = p_{ij}^{\mathrm{R}} \left[1 + m_i (\delta_{c_i} - \delta_{c_j}) + \sum_{k=1}^{C} s_{ij}^{(k)} (\delta_k - \delta_{c_j})\right]. \tag{16}$$

The linearization does not ensure that p_{ij} remains between 0 and 1; thus, we saturate values of $p_{ij} < 0$ and $p_{ij} > 1$ to 0 and 1, respectively.

We provide a simple interpretation for the correction term in (16). The second term in parentheses corrects for the difference in δ values between the origin and destination locations. Should the origin offer worse opportunities than the destination ($\delta_{c_i} > \delta_{c_j}$), this term increases the probability of migration or commuting from i to j (vice versa for $\delta_{c_i} < \delta_{c_j}$). This factor is scaled by the population at i only. Thus, the term modifies the emission from i, such that it is independent of the population at the destination (n_j). The last term in parentheses corrects for the presence of locations of different classes between i and j. If there are locations with better opportunities than the destination ($\delta_k < \delta_{c_j}$), it is more likely for people to stop before reaching j, thus reducing the probability p_{ij}. On the contrary, classes that are worse off than the destination ($\delta_k > \delta_{c_j}$) increase the flux from i to j. This change is modulated by the entire population belonging to each class in a circle of radius r_{ij} around i, excluding populations at i and j.

Draft

Selection of the value of δ-s

The linearized model in (16) offers an indication for the selection of reasonable values of δ-s. We can rewrite the equation as

$$p_{ij}^{(c_i \to c_j)} = p_{ij}^{\mathrm{R}} \left[1 + \sum_{k=1}^{C} s_{ij}^{(k)} (\delta_k - 1) + m_i(\delta_{c_i} - 1) - (m_i + s_{ij})(\delta_{c_j} - 1) \right]. \tag{17}$$

Unlike the nonlinear case, where the probabilities remain always well defined for an arbitrary large value of δ, the probabilities of the linearized model can become negative or larger than one. Thus, there is a limit to the values of δ_{c_i} to guarantee that probabilities are well defined.

From simulations, we find that the main source of ill posedness for the probabilities of the linearized model is becoming larger than one. In fact, the term $\delta_{c_i} - 1$ in each equation is multiplied by the population of some location, such that in real-world datasets even a very small $\delta_{c_i} - 1$ causes sizeable changes in the probabilities. For the probabilities to remain well defined, one could pick $\delta_{c_i} - 1$ to be

$$\delta_{c_i} - 1 \sim (0.1 \div 1) \frac{1}{m_i}. \tag{18}$$

As an indication of the order of magnitude of $\delta - 1$ for the case with two classes, one can use

$$\delta - 1 \sim (0.1 \div 1) \frac{1}{\max_i m_i}. \tag{19}$$

Parameter identification for δ-s

In this manuscript, the parameters δ for the nonlinear model and δ_{c_i} for the linearized model were not fitted, but set to specific values based on order of magnitude arguments detailed in the previous Section. Such an approach allowed us to highlight the potential of our model even without a formal parameter identification technique.

Herein, we propose two parameter identification techniques for δ and δ_{c_i}, which can be used in future efforts to further improve the goodness of fit of the model. For δ, we have a single parameter to identify. In this case, we propose a standard approach based on gradient descent [4]. The algorithm is shown in Algorithm 1. Upon defining the error between real fluxes $\mathbf{T}$ and predicted fluxes $\hat{\mathbf{T}}$ (that depend on the value of δ) in some norm, we consider a gradient descent algorithm to minimize the error. The gradient may be estimated numerically (for example with finite differences) or, in problems of large size, analytically, leveraging

$$\frac{\mathrm{d}E(\delta)}{\mathrm{d}\delta} = \frac{\partial \|\mathbf{T} - \hat{\mathbf{T}}\|}{\partial \hat{\mathbf{T}}} : \frac{\mathrm{d}\hat{\mathbf{T}}}{\mathrm{d}\delta}. \tag{20}$$

In problems of very large size, the parameter α_i may be set equal to a constant from the beginning to avoid evaluating multiple times the error function – at the price of a slower convergence rate. The convergence criteria can include a condition on the norm of E (we stop if the norm of the error goes below a fixed threshold), on the difference $\delta_{i+1} - \delta_i$ (we stop if δ did not change significantly in the last step), and a certain number of iterations (we stop if we repeat the procedures over a certain number of times).

Algorithm 1 Parameter identification for δ

Initialize: $E(\delta) = \|\mathbf{T} - \hat{\mathbf{T}}(\delta)\|$; δ_0; $i = 0$
while not converged **do**
  $g_i = -\left.\frac{\mathrm{d}E(\delta)}{\mathrm{d}\delta}\right|_{\delta_i}$
  $\alpha_i = \arg\min E(\delta_i + \alpha_i g_i)$
  $\delta_{i+1} = \delta_i + \alpha_i g_i$
  $i \leftarrow i + 1$
end while

For δ_{c_i}, we look for parsimonious parameter identification techniques, which only fit some of the most important parameters, while seeking minimal changes in the parameters. The proposed algorithm is in Algorithm 2. In this case, we find the element ij of the error matrix that is largest (in an absolute sense) and identify the class q to which this error is most sensitive (that is, the class with respect to which $\hat{T}_{ij}$ has the largest derivative). This choice allows us to significantly affect the error E_{ij} through a minimal change in δ_q, thus reducing spillover effects on other elements of the error matrix. The value of δ_q is chosen so to minimize the overall error, while keeping the other values of δ_{c_i} constant. To avoid being trapped in a condition where the largest error remains the same (thus always selecting the same q), we exclude the selection of i, j, and q for the next n cycles of the algorithm, where n is a user-defined input that balances between exploration and exploitation. Convergence criteria can be defined in the same way as Algorithm 1.

Draft

Algorithm 2 Parameter identification for δ_{c_i}

Initialize: $E(\delta) = \|\mathbf{T} - \hat{\mathbf{T}}(\delta_1, \ldots, \delta_C)\|$; $\delta_1^0, \ldots, \delta_C^0$; $k = 0$

while not converged **do**

  $i, j = \arg\max \left| T_{ij} - \hat{T}_{ij}(\delta_1^k, \ldots, \delta_C^k) \right|$

  $q = \arg\max \left| s_{ij}^{(q)} + m_i \delta_{c_i q} - (m_i + s_{ij}) \delta_{c_j q} \right|$

  $\alpha_k = \arg\min E(\delta_1^k, \ldots, \delta_q^k + \alpha_k, \ldots, \delta_C^k)$

  $\delta_q^{k+1} = \delta_q^k + \alpha_k$; $\delta_{p \neq q}^{k+1} = \delta_p^k$

  $k \leftarrow k + 1$

  Exclude i, j, q for the next n cycles

end while

Results for South Sudan

The p-values for the MW tests comparing the standard and modified radiation models are shown in Table S1. We find that MW tests are significant for all combinations of variables, provided δ is large enough ($\delta = 1 + 10^{-5}$ or $\delta = 1 + 10^{-4}$). For small values of δ ($1 + 10^{-6}$), only floods and the combination of conflicts and floods leads to significant results. In general, p-values for the modified radiation model based on conflicts only are larger than that of the other two variables, indicating that conflicts improve the prediction of fluxes to a lesser extent than the other two variables. This outcome may be due to the use of casualties over the 2020-2021 period only, which does not account for historical trends of violence over the previous years.

Table S1. p-value of the MW statistical tests for the comparison between the standard and nonlinear modified radiation model with two classes in South Sudan, for each combination of variable of interest and value of n in $\delta = 1 + 10^n$. Results in bold indicate significance ($\alpha = 0.05$).

	-6	-5	-4
Conflicts	0.0505	$<$ **0.0001**	$<$ **0.0001**
Floods	**0.0030**	$<$ **0.0001**	$<$ **0.0001**
Conflicts & Floods	**0.0003**	$<$ **0.0001**	$<$ **0.0001**

We show in Table S2 the z-statistics generated from the MW test [3]. We find that the z-statistics is lower than the 5% percentile of the z-statistics from tests with the random assignment of the class only when considering floods or both conflicts and floods, for sufficiently large values of δ ($1 + 10^{-5}$ and $1 + 10^{-4}$). At small values of δ ($1 + 10^{-6}$), the z-statistics passes the non-parametric test only for the model considering both conflicts and floods. The p-value for non-parametric tests corresponds to the percentile of the corresponding statistics in the surrogate distribution. The p-value for the z-statistics is shown in Table S3. Thus, the improvement in the prediction of fluxes when considering only conflicts (or floods at small δ-s), despite providing p-values that are significant, may be attributed to chance (that is, a better fit of the fluxes with more parameters). On the other hand, fluxes computed accounting for floods at larger δ-s and both conflicts and floods correspond to a genuine improvement of the migration model, better than what could be obtained just by adding more free parameters.

Table S2. z-statistics of the MW statistical tests for the comparison between the standard and nonlinear modified radiation model with two classes in South Sudan, for each combination of variable of interest and value of n in $\delta = 1 + 10^n$. The table also shows the 5% percentile of the empirical distribution of the z-statistics. Results in bold indicate that the z-statistics of the test with a specific variable is lower than the 5% percentile of the empirical distribution of the z-statistics.

	-6	-5	-4
Conflicts	-1.6399	-4.2933	-5.3214
Floods	-2.7442	$\mathbf{-7.0679}$	$\mathbf{-8.6640}$
Conflicts & Floods	$\mathbf{-3.4709}$	$\mathbf{-9.6432}$	$\mathbf{-11.950}$
5% Percentile, Random	-2.7634	-5.8068	-6.9219

Table S3. p-value (that is, percentile in the surrogate distribution) for the non-parametric test based on the z-statistics for the nonlinear modified radiation model with two classes in South Sudan, for each combination of variable of interest and value of n in $\Delta = 10^n$. Results in bold indicate that the z-statistics of the test with a specific variable is lower than the 5% percentile of the empirical distribution of the z-statistics. The p-value resolution is 0.001 (as the surrogate distribution is estimated with 1,000 realizations). The largest value for the percentile based on the resolution is listed in the table, so that the real p-value is equal or less than the one indicated in the table.

	-6	-5	-4
Conflicts	0.511	0.463	0.374
Floods	0.054	**0.002**	**0.001**
Conflicts & Floods	**0.005**	**0.001**	**0.001**

Draft

Results for the statistical tests on Hamming distances are presented in Table S4. We record statistical significance for any variable of interest for any value of δ considered herein.

Table S4. p-value of the statistical tests on Hamming distances for the comparison between the standard and nonlinear modified radiation model with two classes in South Sudan, for each combination of variable of interest and value of n in $\delta = 1 + 10^n$. Results in bold indicate significance ($\alpha = 0.05$).

	-6	-5	-4
Conflicts	$<$ **0.0001**	$<$ **0.0001**	$<$ **0.0001**
Floods	$<$ **0.0001**	$<$ **0.0001**	$<$ **0.0001**
Conflicts & Floods	$<$ **0.0001**	$<$ **0.0001**	$<$ **0.0001**

Table S5 shows the results of the non-parametric test on the empirical distribution of the Hamming distance. p-values for this test are shown in Table S6. For any value of δ and variable of interest, the modified radiation model performs better than the standard one (that is, the Hamming distance is smaller). However, only when considering large enough values of δ ($1 + 10^{-5}$ and $1 + 10^{-4}$) and the effects of floods or both conflicts and floods we obtain results that are below the 5% percentile of the empirical distribution of Hamming distances. At small values of δ ($1 + 10^{-6}$), similar to the z-statistics, only considering both conflicts and floods provides significant results in the non-parametric test. That is, the improvement of the model in the other conditions may only be related to the additional model parameters. Consistently with the MW tests, the modified radiation model that accounts for floods or both conflicts and floods at large enough δ-s represents a genuine improvement in the description of mobility.

Table S5. Hamming distance for the standard and nonlinear modified radiation model with two classes in South Sudan, for each combination of variable of interest and value of n in $\delta = 1 + 10^n$. The table also shows the 5% percentile of the empirical distribution of the Hamming distances. Results in bold indicate that the Hamming distance of the model with a specific variable is lower than the 5% percentile of the empirical distribution of the Hamming distances.

Standard model: 2,718	-6	-5	-4
Conflicts	2,542	2,404	2,364
Floods	2,562	**2,330**	**2,266**
Conflicts & Floods	**2,466**	**2,132**	**2,034**
5% Percentile, Random	2,525	2,366.5	2,327.5

Table S6. p-value (that is, percentile in the surrogate distribution) for the non-parametric test based on the Hamming distance for the nonlinear modified radiation model with two classes in South Sudan, for each combination of variable of interest and value of n in $\Delta = 10^n$. Results in bold indicate that the Hamming distance of the model with a specific variable is lower than the 5% percentile of the empirical distribution of the Hamming distances. The p-value resolution is 0.001 (as the surrogate distribution is estimated with 1,000 realizations). The largest value for the percentile based on the resolution is listed in the table, so that the real p-value is equal or less than the one indicated in the table.

	-6	-5	-4
Conflicts	0.157	0.258	0.239
Floods	0.386	**0.002**	**0.002**
Conflicts & Floods	**0.001**	**0.001**	**0.001**

Numerical comparison of fluxes

Figure S2 shows the comparison of fluxes from standard radiation model and the best performing modified radiation model (floods and conflicts, $\delta = 1 + 10^{-4}$) against real fluxes, in terms of their rank after sorting. While for large fluxes the rank of both models is not well-reconstructed, we find that the modified model remarkably outperforms the standard one, being much closer to the correct rank (the diagonal in Fig. S2) for the majority of the domain. We interpret this result based on the hypotheses underlying our model. In fact, our model penalizes some of the locations, reducing the fluxes that reach them. We do not implement any direct effect on advantageous destination locations, such that larger fluxes may not be accurately reconstructed. On the other hand, we better reconstruct smaller fluxes that have been penalized.

Sensitivity analysis on the penalization threshold

We perform a sensitivity analysis by comparing the results where the 15% of the counties with worst conditions based on conflicts and floods belong to the penalized class against those in which this penalization threshold is 10% and 20%. We repeat the same tests as for the original threshold. Generation of random distributions is repeated due to the different values of the threshold, which affects the number of penalized locations. Comparisons are shown in Tables S7, S8, S9, and S10.

We observe that the results are robust to changes in the penalization threshold, especially for larger values of δ. Apart from obvious differences in numerical results, we record only a few discrepancies in the statistical significance. In the MW statistical

Draft

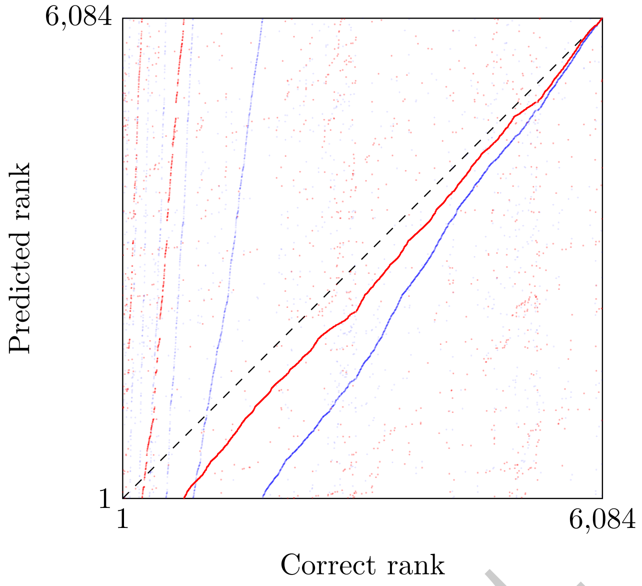

Fig. S2. Comparison of the rank of fluxes of the standard radiation model (blue, circles) and modified radiation model (red, squares) accounting for both floods and conflicts with $\delta = 1 + 10^{-4}$ against the rank of real fluxes. The dashed black line represents a model with zero errors.

test and corresponding non-parametric test, at the lowest value of δ, we find that the modified radiation model with "Floods" as the variable of interest outperforms the standard one when the penalization threshold is 10%, while none of the modified radiation models has a better performance than the standard one for a penalization threshold of 20%. In the Hamming distance test and corresponding non-parametric test, for a threshold of 20%, we barely lose significance for the modified radiation model with "Floods" as the variable of interest.

Table S7. p-value of the MW statistical tests for the comparison between the standard and nonlinear modified radiation model with two classes in South Sudan, for each combination of variable of interest and value of n in $\delta = 1 + 10^n$. Results in bold indicate significance ($\alpha = 0.05$). Different values for the penalization threshold are used.

**Worst** 10%	-6	-5	-4
Conflicts	0.0660	**0.0006**	$<$ **0.0001**
Floods	**0.0021**	$<$ **0.0001**	$<$ **0.0001**
Conflicts & Floods	**0.0002**	$<$ **0.0001**	$<$ **0.0001**
**Worst** 15%	-6	-5	-4
Conflicts	0.0505	$<$ **0.0001**	$<$ **0.0001**
Floods	**0.0030**	$<$ **0.0001**	$<$ **0.0001**
Conflicts & Floods	**0.0003**	$<$ **0.0001**	$<$ **0.0001**
**Worst** 20%	-6	-5	-4
Conflicts	**0.0046**	$<$ **0.0001**	$<$ **0.0001**
Floods	**0.0239**	$<$ **0.0001**	$<$ **0.0001**
Conflicts & Floods	**0.0010**	$<$ **0.0001**	$<$ **0.0001**

Replication of statistical test with Akaike information criterion

To further strengthen our claim that the modified radiation model is a genuine improvement of the standard radiation model, we replicate the statistical test on the Hamming distance through the Akaike information criterion, which explicitly penalizes the number of parameters used in the model [2]. To this end, we consider two likelihood functions (for each variable of interest), corresponding to the null and alternative hypotheses:

- The first likelihood function assumes that the observed Hamming distances H_{std} and H_{mod} arise from a single binomial distribution with success probability p,

$$\mathcal{L}_1(p) = \binom{N^2}{H_{\mathrm{std}}} p^{H_{\mathrm{std}}} (1-p)^{N^2 - H_{\mathrm{std}}} \binom{N^2}{H_{\mathrm{mod}}} p^{H_{\mathrm{mod}}} (1-p)^{N^2 - H_{\mathrm{mod}}}. \tag{21}$$

  Note that the null hypothesis is slightly different from the one tested in the previous section, as the success probability p that minimizes $\mathcal{L}_1(p)$ depends on both standard and modified radiation models.

Draft

Table S8. z-statistics of the MW statistical tests for the comparison between the standard and nonlinear modified radiation model with two classes in South Sudan, for each combination of variable of interest and value of n in $\delta = 1 + 10^n$. The table also shows the 5% percentile of the empirical distribution of the z-statistics. Results in bold indicate that the z-statistics of the test with a specific variable is lower than the 5% percentile of the empirical distribution of the z-statistics. Different values for the penalization threshold are used.

**Worst** 10%	−6	−5	−4
Conflicts	−1.5061	−3.2447	−3.7636
Floods	**−2.8558**	**−5.9966**	**−7.1053**
Conflicts & Floods	**−3.6089**	**−8.0063**	**−9.4367**
5% Percentile, Random	−2.1283	−4.3821	−5.2379
**Worst** 15%	−6	−5	−4
Conflicts	−1.6399	−4.2933	−5.3214
Floods	−2.7442	**−7.0679**	**−8.6640**
Conflicts & Floods	**−3.4709**	**−9.6432**	**−11.950**
5% Percentile, Random	−2.7634	−5.8068	−6.9219
**Worst** 20%	−6	−5	−4
Conflicts	−2.6066	−6.6659	−8.0270
Floods	−1.9800	**−7.3871**	**−9.1374**
Conflicts & Floods	−3.0818	**−10.2565**	**−12.7559**
5% Percentile, Random	−3.4225	−7.1081	−8.6633

Table S9. p-value of the statistical tests on Hamming distances for the comparison between the standard and nonlinear modified radiation model with two classes in South Sudan, for each combination of variable of interest and value of n in $\delta = 1 + 10^n$. Results in bold indicate significance ($\alpha = 0.05$). Different values for the penalization threshold are used.

**Worst** 10%	−6	−5	−4
Conflicts	$<$ **0.0001**	$<$ **0.0001**	$<$ **0.0001**
Floods	$<$ **0.0001**	$<$ **0.0001**	$<$ **0.0001**
Conflicts & Floods	$<$ **0.0001**	$<$ **0.0001**	$<$ **0.0001**
**Worst** 15%	−6	−5	−4
Conflicts	$<$ **0.0001**	$<$ **0.0001**	$<$ **0.0001**
Floods	$<$ **0.0001**	$<$ **0.0001**	$<$ **0.0001**
Conflicts & Floods	$<$ **0.0001**	$<$ **0.0001**	$<$ **0.0001**
**Worst** 20%	−6	−5	−4
Conflicts	$<$ **0.0001**	$<$ **0.0001**	$<$ **0.0001**
Floods	$<$ **0.0001**	$<$ **0.0001**	$<$ **0.0001**
Conflicts & Floods	$<$ **0.0001**	$<$ **0.0001**	$<$ **0.0001**

- The second likelihood function assumes that the observed Hamming distances H_{std} and H_{mod} arise from two different binomial distribution with success probabilities p_1 and p_2,

$$\mathcal{L}_2(p_1, p_2) = \binom{N^2}{H_{\text{std}}} p_1^{H_{\text{std}}} (1-p_1)^{N^2 - H_{\text{std}}} \binom{N^2}{H_{\text{mod}}} p_2^{H_{\text{mod}}} (1-p_2)^{N^2 - H_{\text{mod}}}. \tag{22}$$

We then compute the Akaike information criterion (AIC) for each of these models as

$$\text{AIC}_i = 2k_i - 2\log\left(\hat{\mathcal{L}}_i\right), \tag{23}$$

where $i = 1$ or 2, k_i is the number of parameters of model i, and $\hat{\mathcal{L}}_i$ is the maximum likelihood value for model i as a function of its parameters. We assign $k_1 = 1$ (p only) for the first likelihood function and $k_2 = 4$ (p_1, p_2, δ, and the percentile of the distribution of variables that separates the two classes of locations). Note that we only run the optimization for $\mathcal{L}_2$ over p_1 and p_2, such that the AIC found herein is an overestimate of the actual AIC (such that it represents a conservative estimate). Such a decision is related to the complexity of the optimization with respect to δ and the percentile of the distribution of variables that separates the two classes of locations, which are selected empirically.

Finally, we compute the relative likelihood of the two models as

$$\text{RL}_i = \exp\left(\frac{\min_j \text{AIC}_j - \text{AIC}_i}{2}\right), \tag{24}$$

which is proportional to the probability that model i minimizes information loss. The model that most probably minimizes information loss will have RL = 1, while the other will have RL $<$ 1. A smaller value of the relative likelihood indicates a model that is more unlikely to correctly represent the information contained in the data.

Results for the relative likelihoods are shown in Tab. S11. For large enough values of δ ($1+10^{-5}$ and $1+10^{-4}$), all combinations of variables yield a model that is at least 10,000 times more likely to minimize information loss compared to the standard radiation

Draft

Table S10. Hamming distance for the standard and nonlinear modified radiation model with two classes in South Sudan, for each combination of variable of interest and value of n in $\delta = 1 + 10^n$. The table also shows the 5% percentile of the empirical distribution of the Hamming distances. Results in bold indicate that the Hamming distance of the model with a specific variable is lower than the 5% percentile of the empirical distribution of the Hamming distances. Different values for the penalization threshold are used. The Hamming distance for the standard model is 2,718.

**Worst** 10%	−6	−5	−4
Conflicts	2,573	2,486	2,463
Floods	2,569	**2,401**	**2,358**
Conflicts & Floods	**2,498**	**2,269**	**2,210**
5% Percentile, Random	2,569	2,467	2,429.5
**Worst** 15%	−6	−5	−4
Conflicts	2,542	2,404	2,364
Floods	2,562	**2,330**	**2,266**
Conflicts & Floods	**2,466**	**2,132**	**2,034**
5% Percentile, Random	2,525	2,366.5	2,327.5
**Worst** 20%	−6	−5	−4
Conflicts	2,533	2,306	2,251
Floods	2,573	2,280	**2,205**
Conflicts & Floods	**2,474**	**2,072**	**1,961**
5% Percentile, Random	2,481	2,277	2,220

model. Interestingly, the modified radiation model that only considers conflicts is over 10^7 times more likely than the standard one to minimize information loss at large values of δ $(1 + 10^{-4})$, in contrast with previous results, suggesting that the non-parametric test may be conservative. These results further confirm that the improvement in the predictions in the modified radiation model is not only related to the additional number of parameters used.

Table S11. Relative likelihood for the standard (RL_1) and modified radiation model (RL_2), for the Hamming distance test in South Sudan, with different variables used for the selection of classes and values of n in $\delta = 1 + 10^n$. Results in bold indicate instances in which the standard radiation model is less than 5% likely to minimize information loss compared to the modified radiation model.

		−6	−5	−4
Conflicts	RL_1	0.1122	$\mathbf{1.2042 \times 10^{-6}}$	$\mathbf{1.2650 \times 10^{-8}}$
	RL_2	1	1	1
Floods	RL_1	0.3425	$\mathbf{1.6866 \times 10^{-10}}$	$\mathbf{1.6213 \times 10^{-14}}$
	RL_2	1	1	1
Conflicts & Floods	RL_1	$\mathbf{4.6439 \times 10^{-4}}$	$\mathbf{4.8977 \times 10^{-25}}$	$\mathbf{1.3507 \times 10^{-34}}$
	RL_2	1	1	1

Statistical tests with weighted Hamming distance and F_1-scores

To study how the model reconstructs non-zero fluxes, we consider two additional measures. First, we examine the weighted Hamming distance, using as a weight the absolute error itself, such that the weighted Hamming distance corresponds to the 1-norm of the error matrix. Second, we investigate the F_1-score (that is, the harmonic mean of precision and recall) [1], based on whether fluxes are computed correctly or not.

We first consider the weighted Hamming distance. Extending the statistical test on the standard Hamming distance based on the binomial distribution to the weighted Hamming distance is not computationally feasible. In fact, this test would require estimating the cumulative mass function of a multinomial distribution, which would call for computations that scale combinatorially with the number of counties. Thus, we limit ourselves to the comparison of the weighted Hamming distance between standard and modified radiation models, as well as the non-parametric test based on the surrogate distribution of the weighted Hamming distance.

Results for the weighted Hamming distance are shown in Table S12. We find that none of the weighted Hamming distances estimated for our model are lower than either the standard radiation model or the 5% percentile of the empirical distribution of the weighted Hamming distance (as confirmed by the p-values of the non-parameteric test, as shown in Table S13). This result shows that neither the standard nor the modified radiation model are able to capture larger migration fluxes in South Sudan. Likely, local factors that are not accounted for in either model shape the largest migration patterns.

Next, we consider the F_1-score for whether fluxes are correctly reconstructed or not. The F_1-score is computed from true positive (TP), false negative (FN), and false positive (FP) results as

$$F_1 = \frac{2\,\mathrm{TP}}{2\,\mathrm{TP} + \mathrm{FP} + \mathrm{FN}}. \tag{25}$$

The results for the F_1-score is shown in Table S14. The F_1-scores for all conditions are better than that of the standard model. In this case, we obtain the same results as for the MW-test and Hamming distance in terms of the non-parametric test on the

Draft

Table S12. Weighted Hamming distance for the standard and nonlinear modified radiation model with two classes in South Sudan, for each combination of variable of interest and value of n in $\delta = 1 + 10^n$. The table also shows the 5% percentile of the empirical distribution of the weighted Hamming distances. Results in bold indicate that the weighted Hamming distance of the model with a specific variable is lower than the 5% percentile of the empirical distribution of the weighted Hamming distances.

Standard model: 224,694	−6	−5	−4
Conflicts	226,527	230,953	231,839
Floods	227,128	232,659	234,463
Conflicts & Floods	226,234	236,836	239,445
5% Percentile, Random	221,430	219,746	222,415.5

Table S13. p-value (that is, percentile in the surrogate distribution) for the non-parametric test based on the weighted Hamming distance for the nonlinear modified radiation model with two classes in South Sudan, for each combination of variable of interest and value of n in $\Delta = 10^n$. Results in bold indicate that the weighted Hamming distance of the model with a specific variable is lower than the 5% percentile of the empirical distribution of the weighted Hamming distances. The p-value resolution is 0.001 (as the surrogate distribution is estimated with 1,000 realizations). The largest value for the percentile based on the resolution is listed in the table, so that the real p-value is equal or less than the one indicated in the table.

	−6	−5	−4
Conflicts	0.556	0.409	0.336
Floods	0.651	0.501	0.422
Conflicts & Floods	0.533	0.638	0.574

surrogate distribution of F_1-scores, as confirmed in Table S15. This result confirms the ability of the proposed model to better capture the statistical distribution of migration patterns.

Table S14. F_1-score for the standard and nonlinear modified radiation model with two classes in South Sudan, for each combination of variable of interest and value of n in $\delta = 1 + 10^n$. The table also shows the 95% percentile of the empirical distribution of the F_1-scores. Results in bold indicate that the F_1-score of the model with a specific variable is higher than the 95% percentile of the empirical distribution of the F_1-scores.

Standard model: 0.7124	−6	−5	−4
Conflicts	0.7359	0.7538	0.7589
Floods	0.7333	**0.7632**	**0.7712**
Conflicts & Floods	**0.7458**	**0.7876**	**0.7993**
95% Percentile, Random	0.7380	0.7582	0.7637

Table S15. p-value (that is, percentile in the surrogate distribution) for the non-parametric test based on the F_1-score for the nonlinear modified radiation model with two classes in South Sudan, for each combination of variable of interest and value of n in $\Delta = 10^n$. Results in bold indicate that the F_1-score of the model with a specific variable is higher than the 95% percentile of the empirical distribution of the F_1-scores. The p-value resolution is 0.001 (as the surrogate distribution is estimated with 1,000 realizations). The largest value for the percentile based on the resolution is listed in the table, so that the real p-value is equal or less than the one indicated in the table.

	−6	−5	−4
Conflicts	0.154	0.242	0.243
Floods	0.367	**0.003**	**0.001**
Conflicts & Floods	**0.001**	**0.001**	**0.001**

Spearman correlation analysis

To complement the previous tests, we compute the Spearman correlation coefficient between real fluxes and the fluxes predicted by the standard and modified radiation models. Results are shown in Table S16. For South Sudan, we find that the Spearman correlation coefficient between real fluxes and fluxes from the standard radiation model is always slightly higher than that between real fluxes and fluxes from the modified radiation model, regardless of Δ and the variable of interest. While the modified model provides a better representation of the statistical distribution of fluxes, it is unable (similar to the standard radiation model) to fully capture the decision-making process that underlies strong migration patterns caused by a mix of civil war and flooding. This limitation may explain the lack of improvements in both the weighted Hamming distance and Spearman correlation coefficient.

Draft

Table S16. Spearman correlation coefficient for the standard and nonlinear modified radiation model with two classes in South Sudan, for each combination of variable of interest and value of n in $\Delta = 10^n$.

Standard model: 0.3600	−6	−5	−4
Conflicts	0.3434	0.3162	0.3063
Floods	0.3534	0.3472	0.3491
Conflicts & Floods	0.3322	0.2946	0.2885

Plots of variables of interests in the United States

Figure S3 shows the counties in the US that have a value of each variable of interest (Gini index, poverty ratio, ratio between the median rent and median household income, and unemployment rate) over the 90% percentile.

Results for the United States

We show the p-values for the MW tests comparing the standard and modified radiation models in Table S17. For moderate values of Δ (10^{-7}), all variables of interests used in the modified radiation models yield statistical significance. For smaller values of Δ (10^{-8}), we only register a significant result for the ratio between median rent and median income. At higher Δ values, only modified radiation models based on poverty ratio and unemployment rate outperform the standard radiation model. We interpret the lack of significance at small values of Δ as an effect of the socioeconomic variables which is too weak to generate substantial changes in the fluxes. On the other hand, for larger values of Δ, the rejection of higher-order terms in the linearized model is likely to generate large errors (note that δ values are multiplied by large coefficients, so that even small variations of δ from 1 may cause large errors compared to the nonlinear model).

Table S17. p-value of the MW statistical tests for the comparison between the standard and linearized modified radiation model with arbitrary number of classes in the US, for each combination of variable of interest and value of n in $\Delta = 10^n$. Results in bold indicate significance ($\alpha = 0.05$).

	−8	−7	−6
Gini index	0.1045	< **0.0001**	0.8683
Poverty ratio	0.0652	< **0.0001**	< **0.0001**
Median rent/household income	**0.0288**	< **0.0001**	1
Unemployment rate	0.2358	< **0.0001**	< **0.0001**

Results for the z-statistics of the MW tests are shown in Table S18. The corresponding p-value of the non-parametric test is in Table S19. For small values of Δ (10^{-8}), the z-statistics for all variables is lower than the 5% percentile of the empirical distribution. Similarly, at moderate values of Δ (10^{-7}), we find results below the 5% percentile for all variables but the unemployment rate. In these conditions, the proposed model genuinely outperforms the standard radiation model. On the other hand, at larger values of Δ (10^{-6}), all improvements can be traced back to the larger number of parameters in the model.

Table S18. z-statistics of the MW statistical tests for the comparison between the standard and linearized modified radiation model with arbitrary number of classes in the US, for each combination of variable of interest and value of n in $\Delta = 10^n$. The table also shows the 5% percentile of the empirical distribution of the z-statistics. Results in bold indicate that the z-statistics of the test with a specific variable is lower than the 5% percentile of the empirical distribution of the z-statistics.

	−8	−7	−6
Gini index	**−1.2562**	**−6.4471**	1.1187
Poverty ratio	**−1.5127**	**−7.8541**	−15.555
Median rent/household income	**−1.8990**	**−6.7372**	5.6317
Unemployment rate	**−0.7200**	−4.2564	−11.871
5% Percentile, Random	−0.6783	−5.9074	−16.042

Table S20 shows the p-values of the statistical tests on Hamming distances for US commuting patterns, comparing standard and linearized modified radiation models. Regardless of the value of Δ, we register statistically significant differences between the models, apart for a single condition (unemployment ratio with $\Delta = 10^{-8}$). Results on the non-parametric test on Hamming distances are displayed in Table S21, with corresponding p-values in Table S22. The Hamming distance for the standard radiation model is higher than that for any modified radiation model. At low and moderate values of Δ (10^{-8} and 10^{-7}), we find that the Hamming distance is below the 5% percentile of the empirical distribution for any variable (apart for unemployment ratio with $\Delta = 10^{-7}$). Therefore, in all of these conditions, the modified radiation model performs better than just fitting with additional model parameters. For larger values of Δ (10^{-6}), none of the proposed models rejects the null of the non-parametric test. In the cases in which the non-parametric test fails, we attribute the improvements in the predictions of fluxes to the additional model parameters.

Draft

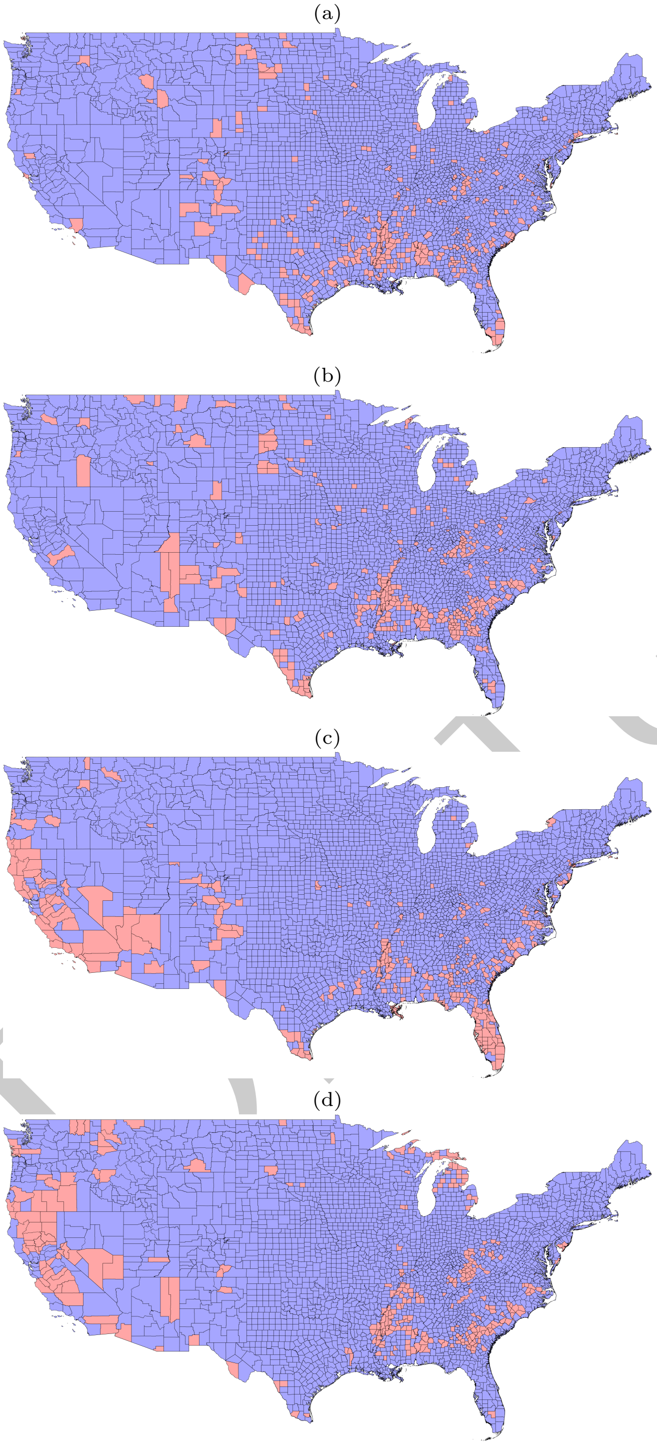

Fig. S3. US counties (red) with the largest 10% values of: (a) Gini index; (b) poverty ratio; (c) ratio between the median rent and median household income; and (d) unemployment rate.

Statistical tests with weighted Hamming distance and F_1-scores

Similar to the case of South Sudan, we introduce statistical tests on the weighted Hamming distance and F_1-scores for the US case study. Results on the non-parametric test on the weighted Hamming distance are listed in Table S23, with corresponding p-values in Table S24. We find that poverty ratio and unemployment rate are the only variables that generate models with lower weighted Hamming distances than the standard radiation model, and at the same time reject the null of the non-parametric test. This result confirms the importance of accounting for poverty ratio in determining commuting fluxes, and points at more complex effects of the unemployment rate on commuting that is not captured by other tests.

Table S19. p-value (that is, percentile in the surrogate distribution) for the non-parametric test based on the z-statistics for the linearized modified radiation model with arbitrary number of classes in the US, for each combination of variable of interest and value of n in $\Delta = 10^n$. Results in bold indicate that the z-statistics of the test with a specific variable is lower than the 5% percentile of the empirical distribution of the z-statistics. The p-value resolution is 0.005 (as the surrogate distribution is estimated with 200 realizations). The largest value for the percentile based on the resolution is listed in the table, so that the real p-value is equal or less than the one indicated in the table.

	−8	−7	−6
Gini index	**0.005**	**0.005**	1
Poverty ratio	**0.005**	**0.005**	0.185
Median rent/household income	**0.005**	**0.005**	1
Unemployment rate	**0.015**	0.965	0.965

Table S20. p-value of the statistical tests on Hamming distances for the comparison between the standard and linearized modified radiation model with arbitrary number of classes in the US, for each combination of variable of interest and value of n in $\Delta = 10^n$. Results in bold indicate significance ($\alpha = 0.05$).

	−8	−7	−6
Gini index	**0.0278**	$<$ **0.0001**	$<$ **0.0001**
Poverty ratio	**0.0247**	$<$ **0.0001**	$<$ **0.0001**
Median rent/household income	**0.0035**	$<$ **0.0001**	$<$ **0.0001**
Unemployment rate	0.1641	$<$ **0.0001**	$<$ **0.0001**

Table S21. Hamming distance for the standard and linearized modified radiation model with arbitrary number of classes in the US, for each combination of variable of interest and value of n in $\Delta = 10^n$. The table also shows the 5% percentile of the empirical distribution of Hamming distances. Results in bold indicate that the Hamming distance of the model with a specific variable is lower than the 5% percentile of the empirical distribution of the Hamming distances.

Standard model: 341,853	−8	−7	−6
Gini index	**340,754**	**336,052**	337,443
Poverty ratio	**340,724**	**336,051**	330,436
Median rent/household income	**340,306**	**335,676**	338,684
Unemployment rate	**341,291**	338,746	333,428
5% Percentile, Random	341,347.5	337,418.5	330,047

Table S22. p-value (that is, percentile in the surrogate distribution) for the non-parametric test based on the Hamming distance for the linearized modified radiation model with arbitrary number of classes in the US, for each combination of variable of interest and value of n in $\Delta = 10^n$. Results in bold indicate that the Hamming distance of the model with a specific variable is lower than the 5% percentile of the empirical distribution of the Hamming distances. The p-value resolution is 0.005 (as the surrogate distribution is estimated with 200 realizations). The largest value for the percentile based on the resolution is listed in the table, so that the real p-value is equal or less than the one indicated in the table.

	−8	−7	−6
Gini index	**0.005**	**0.005**	1
Poverty ratio	**0.005**	**0.005**	0.175
Median rent/household income	**0.005**	**0.005**	1
Unemployment rate	**0.005**	0.990	0.995

Table S23. Weighted Hamming distance for the standard and linearized modified radiation model with arbitrary number of classes in the US, for each combination of variable of interest and value of n in $\Delta = 10^n$. The table also shows the 5% percentile of the empirical distribution of weighted Hamming distances. Results in bold indicate that the Hamming distance of the model with a specific variable is lower than the 5% percentile of the empirical distribution of the weighted Hamming distances.

Standard model: 33,211,513	−8	−7	−6
Gini index	33,222,536	33,311,399	34,520,101
Poverty ratio	**33,208,043**	**33,194,116**	33,212,251
Median rent/household income	33,217,744	33,287,913	34,444,204
Unemployment rate	**33,208,174**	**33,189,546**	34,444,204
5% Percentile, Random	33,209,407.5	33,194,479.5	33,155,470.5

The F_1-score for the models for the US are shown in Table S25. The p-values of the corresponding non-parametric test are listed in Table S26. In this case, the $F1$-score is much larger than for South Sudan, indicating that both models are able to capture most of the fluxes. Regardless of the value of Δ and of the variable of interest, all modified models have a F_1-score that is higher than the standard model. All variables at $\Delta = 10^{-8}$ and $\Delta = 10^{-7}$ (apart from the unemployment rate) pass the non-parametric test, providing results that are consistent with the standard Hamming distance.

Draft

Table S24. p-value (that is, percentile in the surrogate distribution) for the non-parametric test based on the weighted Hamming distance for the linearized modified radiation model with arbitrary number of classes in the US, for each combination of variable of interest and value of n in $\Delta = 10^n$. Results in bold indicate that the weighted Hamming distance of the model with a specific variable is lower than the 5% percentile of the empirical distribution of the weighted Hamming distances. The p-value resolution is 0.005 (as the surrogate distribution is estimated with 200 realizations). The largest value for the percentile based on the resolution is listed in the table, so that the real p-value is equal or less than the one indicated in the table.

	−8	−7	−6
Gini index	1	1	1
Poverty ratio	**0.005**	**0.035**	0.670
Median rent/household income	1	1	1
Unemployment rate	**0.005**	**0.005**	0.075

Table S25. F_1-score for the standard and linearized modified radiation model with arbitrary number of classes in the US, for each combination of variable of interest and value of n in $\Delta = 10^n$. The table also shows the 95% percentile of the empirical distribution of F_1-scores. Results in bold indicate that the F_1-score of the model with a specific variable is higher than the 95% percentile of the empirical distribution of the F_1-scores.

Standard model: 0.98199	−8	−7	−6
Gini index	**0.98205**	**0.98230**	0.98222
Poverty ratio	**0.98205**	**0.98230**	0.98260
Median rent/household income	**0.98207**	**0.98232**	0.98216
Unemployment rate	**0.98202**	0.98215	0.98244
95% Percentile, Random	0.98201	0.98222	0.98262

Table S26. p-value (that is, percentile in the surrogate distribution) for the non-parametric test based on the F_1-score for the linearized modified radiation model with arbitrary number of classes in the US, for each combination of variable of interest and value of n in $\Delta = 10^n$. Results in bold indicate that the F_1-score of the model with a specific variable is higher than the 95% percentile of the empirical distribution of the F_1-scores. The p-value resolution is 0.005 (as the surrogate distribution is estimated with 200 realizations). The largest value for the percentile based on the resolution is listed in the table, so that the real p-value is equal or less than the one indicated in the table.

	−8	−7	−6
Gini index	**0.005**	**0.005**	1
Poverty ratio	**0.005**	**0.005**	0.175
Median rent/household income	**0.005**	**0.005**	1
Unemployment rate	**0.005**	0.990	0.995

Spearman correlation analysis

Similar to South Sudan, we perform a Spearman correlation analysis for the US commuting patterns (between real fluxes and fluxes predicted by models). To limit the computational burden, we compute the Spearman correlation coefficient only for the elements that, for each Δ, are estimated to be non-zero by at least one model or are non-zero in the real commuting data. This approach is chosen so that the Spearman correlation coefficients are computed for arrays with the same size, making their comparison fair. Results are shown in Table S27. For all values of Δ, the modified radiation model using poverty ratio and unemployment rate have a Spearman correlation coefficient that is higher than that of the standard model. This result is analogous to that of the weighted Hamming distance, confirming the ability of the modified radiation model to outperform the standard radiation model based on a variety of performance metrics.

Table S27. Spearman correlation coefficient for the standard and linearized modified radiation models with arbitrary number of classes in the US, for each combination of variable of interest and value of n in $\Delta = 10^n$. A value is provided for the standard model at each Δ, as a different set of elements is used to compute the correlation coefficient.

	−8	−7	−6
Gini index	0.1305	0.1198	0.1003
Poverty ratio	0.1349	0.1437	0.1656
Median rent/household income	0.1308	0.1260	0.1086
Unemployment rate	0.1339	0.1413	0.1669
Standard model	0.1329	0.1363	0.1594

References

1. Tom Fawcett. An introduction to ROC analysis. *Pattern Recognition Letters*, 27(8):861–874, 2006.
2. Sadanori Konishi and Genshiro Kitagawa. *Information criteria and statistical modeling.* Springer Science & Business Media, New York, New York, United States, 2008.

Draft

3. Mathworks. ranksum. https://www.mathworks.com/help/stats/ranksum.html, accessed: 06/2024, 2024.
4. Kevin P Murphy. *Probabilistic machine learning: an introduction*. MIT press, 2022.

Draft
